# The organelle oxidative-inflammatory network in polycystic ovary syndrome: from subcellular intercellular cascades to systemic comorbidities

**DOI:** 10.3389/fimmu.2026.1922372

**Published:** 2026-09-23

**Authors:** Kaiqing Ou, Dezhi Zhang, Yuanpeng Liao, Ting Yu, Wenxing Liu, Ting Sun, Ying Huang

**Affiliations:** 1 School of Traditional Chinese Medicine, Hubei University of Chinese Medicine, Wuhan, China; 2 Jingzhou Hospital of Traditional Chinese Medicine, Affiliated Hospital of Hubei University of Chinese Medicine, Jingzhou, China; 3 Department of Pharmacology, School of Medicine, Yangtze University, Jingzhou, China

**Keywords:** polycystic ovary syndrome (PCOS), systemic comorbidities, cardiometabolic complications, organelle homeostasis, oxidative-inflammation network, targeted therapy

## Abstract

Polycystic ovary syndrome (PCOS) transcends localized reproductive dysfunction, manifesting as a systemic metabolic disorder driven primarily by insulin resistance (IR) and chronic inflammation. The condition was recently redesignated as polyendocrinometabolic syndrome (PMOS) to better reflect this systemic pathophysiology. (Note: This review retains the traditional PCOS nomenclature to maintain consistency with existing literature.) This review identifies the organelle oxidative network, characterized by mitochondrial dysfunction and endoplasmic reticulum (ER) stress, as a critical pathological nexus in PCOS progression. Lipotoxicity and glucotoxicity disrupt organelle homeostasis, precipitating macrophage metabolic reprogramming and diverse programmed cell death pathways, including ferroptosis and pyroptosis. Furthermore, the release of organelle-derived damage-associated molecular patterns (DAMPs) and extracellular vesicles amplifies localized metabolic insults into systemic cascades, exacerbating cardiovascular and glucolipid complications. We conclude by evaluating the therapeutic potential of targeting organelle defense systems and dissecting the deep cellular mechanisms of current metabolic agents, establishing a novel framework for PCOS diagnosis and clinical translation.

## 1 Introduction

Polycystic ovary syndrome (PCOS) is the most prevalent reproductive and endocrinometabolic disorder among women of reproductive age (1). Because this condition extends beyond localized reproductive dysfunction to manifest as a lifelong systemic metabolic syndrome, an international consortium recently redesignated it as polyendocrinometabolic syndrome (PMOS) (2). Accordingly, this nomenclature update supports our central thesis that these intertwined endocrinometabolic perturbations are systemically associated with insulin resistance (IR) and chronic inflammation. Nevertheless, to ensure consistency with existing literature, this article retains he traditional PCOS acronym. Core clinical hallmarks include ovulatory dysfunction, polycystic ovarian morphology, and hyperandrogenism (3), collectively contributing to abnormal folliculogenesis and anovulatory infertility (4). However, PCOS extends beyond localized reproductive lesions, manifesting as a lifelong systemic metabolic syndrome (5). These intertwined endocrine and metabolic perturbations are closely linked to systemic IR and chronic inflammation (6, 7). Disrupted physiological homeostasis (8) significantly elevates the long-term risk of cardiovascular disease, atherosclerosis, and type 2 diabetes (9). This metabolically toxic microenvironment contributes to neuropsychiatric anomalies and cognitive impairment in premenopausal women (10), underscoring the imperative for early systemic intervention.

Pathophysiologically, hyperandrogenism and low-grade chronic inflammation are among the key factors implicated in PCOS progression (11). Hyperandrogenism promotes cytokine hypersecretion, acting as a critical determinant of sex-specific cardiovascular risk (12, 13). It tissue-specifically remodels reproductive, metabolic, and immune microenvironments (14), synergizing with systemic inflammation to facilitate substantial follicular hypoplasia (15). Peripheral inflammatory mediators infiltrate the follicular microenvironment, initiating a substantial local inflammatory cascade (16). Metabolic toxicity amplifies this pathology. Elevated palmitic acid from excessive saturated fats triggers innate immunity, linking local reproductive defects to distal glucolipid comorbidities (17, 18). Furthermore, reproductive-endocrine accumulation of purine metabolites, notably uric acid, exacerbates systemic inflammation (19).

Multi-omics advancements have shifted PCOS research from macroscopic endocrine imbalances toward microscopic organelle-immune interaction networks (20). Transcriptomic, proteomic, and endometrial molecular profiling consistently implicate micro-molecular dysregulation in disease pathogenesis (21–23). Therefore, we propose that a peroxidation network integrating mitochondrial dysfunction, endoplasmic reticulum (ER) stress, and chronic inflammation acts as the central pathological node in PCOS progression (24). Within this network, diminished antioxidant capacity and excessive reactive oxygen species (ROS) elicit substantial systemic oxidative and nitrosative stress (25, 26). This organelle-mediated oxidative nexus plays a pivotal role in the multidimensional pathologies and infertility characteristic of PCOS (27).

Structurally, this oxidative-inflammatory network couples mitochondria, the ER, and lysosomes or autophagosomes. Molecularly, it exhibits robust synergy between excessive ROS and pro-inflammatory mediators, notably the NLRP3 inflammasome (28, 29). This mitochondria-centric cascade propagates oxidative damage to the nucleus and extracellular microenvironment, facilitating substantial inflammation (30, 31). Under pathological stress, impaired negative feedback mechanisms, specifically the NRF2 pathway, predispose this network to a vicious cycle of ROS accumulation, immune hyperactivation, and progressive oxidative injury (32, 33). Within the reproductive tract, this network localizes to oocytes, granulosa cells, endometrial cells, and resident immune populations at the maternal-fetal interface (27, 34). Although basal oxidation and inflammation maintain reproductive homeostasis, their dysregulation is strongly associated with PCOS, follicular arrest, and adverse pregnancy outcomes. Thus, organelle-targeted antioxidant and anti-inflammatory therapies represent a highly promising clinical frontier (35, 36).

This oxidative-inflammatory microenvironment disrupts organelle homeostasis, contributing to complex inter-organ damage and non-apoptotic programmed cell death. Ferroptosis, mediated by iron overload and lipid peroxidation, significantly contributes to impaired endometrial receptivity and granulosa cell damage (37). Autophagic dysregulation and its immune cross-talk form a central hub within the liver-adipose-ovary axis, disrupting reproductive micro-remodeling (38, 39). This inter-organ dialogue is particularly evident in the ovary-liver axis, where bidirectional dysregulation of lipid homeostasis and reproductive endocrinology renders non-alcoholic fatty liver disease (NAFLD) a prevalent systemic comorbidity of PCOS (40). Circadian rhythm disruption (41), excessive mTORC1 activation (42), and aberrant TET2-mediated epigenetic modifications (43) synergistically govern this peroxidation-inflammation network.

PCOS pathogenesis extends beyond reproductive dysfunction to encompass a systemic network of metabolic toxicity, organelle damage, and immune dysregulation (29). Centering on the oxidation-inflammation nexus, this review introduces two novel perspectives to unravel these complex pathological mechanisms. First, rather than treating oxidative stress, organelle dysfunction, and inflammation as isolated pathways, we conceptualize them as an integrated organelle oxidative-inflammatory network. Within this framework, we systematically delineate how lipotoxicity and glucotoxicity perturb mitochondrial and ER homeostasis, thereby mediating macrophage reprogramming and programmed cell death cascades, including apoptosis (44). Second, organelle-derived damage-associated molecular patterns (DAMPs) and extracellular vesicles propagate local ovarian metabolic insults into systemic glucolipid, hepatic, and cardiovascular comorbidities. We evaluate the therapeutic potential of targeting organelle defense systems, skeletal muscle factors (45), and novel metabolic biomarkers such as sex hormone-binding globulin (SHBG) for precision multi-target interventions (46). Ultimately, unifying subcellular interplay with systemic outcomes establishes an innovative theoretical framework to advance the comprehensive diagnosis, clinical translation, and management of PCOS.

## 2 Metabolic toxicity-mediated disruption of organelle network homeostasis

Extending beyond localized reproductive dysfunction, PCOS constitutes a systemic syndrome mediated by upstream metabolic toxicity. Chronic glucolipid overload contributes to global organelle dyshomeostasis. Serving as a crucial intermediate mediator, this subcellular dysfunction translates metabolic insults into substantial cellular damage. Combined glucolipotoxicity structurally and functionally disrupts mitochondrial networks, promotes chronic ER stress, and impairs lysosomal-autophagic machinery (**Figure 1**).

**Figure 1:**
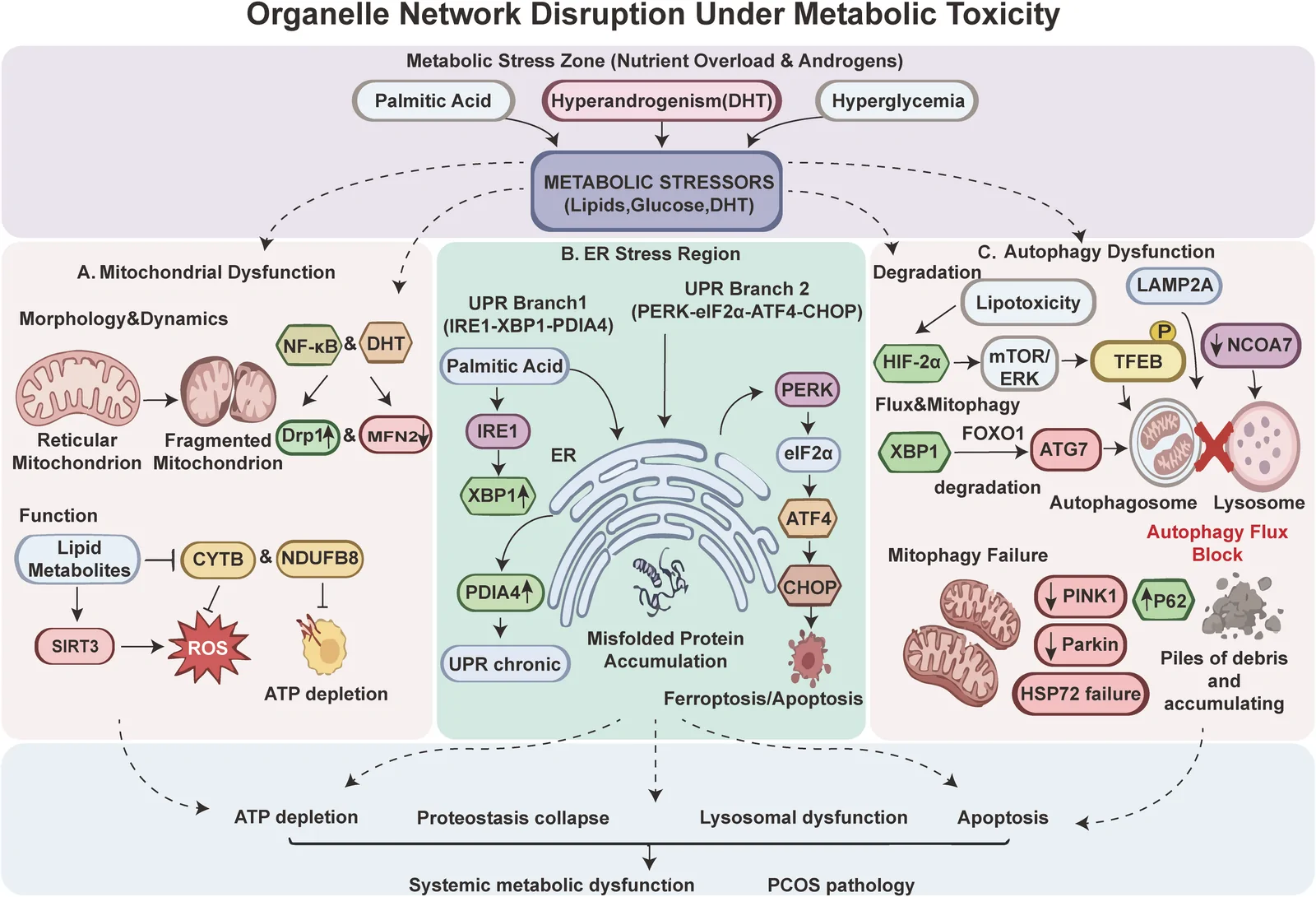
Metabolic toxicity- mediated organelle dyshomeostasis. Hyperglycemia, palmitate, and DHT constitute major metabolic stressors facilitating the organelle damage cascade within the PCOS microenvironment. **(A)** Mitochondrial dysfunction. NF-κB activation and DHT upregulate DRP1 and downregulate MFN2 to promote marked mitochondrial fragmentation. Lipid metabolites inhibit respiratory proteins CYTB and NDUFB8, which, alongside SIRT3 downregulation, contributes to substantial ROS accumulation and ATP reduction. **(B)** ER stress. Metabolic overload accumulates misfolded proteins to activate the UPR. Palmitate activates the IRE1-XBP1-PDIA4 axis to mediate chronic ER stress, whereas PERK-eIF2α-ATF4-CHOP hyperactivation increases cellular susceptibility to ferroptosis and apoptosis. **(C)** Autophagic dysfunction. Lipotoxicity inactivates TFEB via HIF-2α and mTOR/ERK pathways, which alongside LAMP2A upregulation and NCOA7 deficiency substantially impairs lysosomal degradation.XBP1 overexpression degrades FOXO1 and depletes ATG7 to substantially impede autophagosome-lysosome fusion. Deleterious debris including P62 and damaged mitochondria accumulate substantially, further exacerbated by defective mitophagy stemming from PINK1/PARKIN downregulation and HSP72 dysfunction. Ultimately, the physical and functional disruption of these interconnected organelles contributes to ATP reduction, proteostasis dysfunction, lysosomal impairment, and apoptosis, establishing the micropathological foundation for systemic metabolic dysfunction in PCOS. PCOS, Polycystic Ovary Syndrome; DHT, Dihydrotestosterone; ROS, Reactive Oxygen Species; ATP, Adenosine Triphosphate; NF-κB, Nuclear Factor kappa-light-chain-enhancer of activated B cells; DRP1, Dynamin-related protein 1; MFN2, Mitofusin-2; CYTB, Cytochrome b; NDUFB8, NADH, ubiquinone oxidoreductase subunit B8;SIRT3, Sirtuin 3;ER, Endoplasmic Reticulum; UPR, Unfolded Protein Response; IRE1, Inositol-requiring enzyme 1; XBP1, X-box binding protein 1; PDIA4, Protein disulfide isomerase family A member 4; PERK, Protein kinase RNA-like endoplasmic reticulum kinase; eIF2α, Eukaryotic initiation factor 2 alpha; ATF4, Activating transcription factor 4; CHOP, C/EBP homologous protein; HIF-2α, Hypoxia-inducible factor 2-alpha; mTOR, Mammalian target of rapamycin; ERK, Extracellular signal-regulated kinase; TFEB, Transcription factor EB; LAMP2A, Lysosome-associated membrane protein 2A; NCOA7, Nuclear receptor coactivator 7; FOXO1, Forkhead box protein O1;ATG7, Autophagy related 7; PINK1, PTEN-induced kinase 1; HSP72, Heat shock protein 72.

### 2.1 Disruption of the mitochondrial network by lipotoxicity/glucotoxicity

PCOS extends beyond reproductive endocrine dysfunction; its progression is significantly promoted by a glucolipotoxic microenvironment characterized by hyperglycemia and elevated free fatty acids. Continuous glucolipotoxicity substantially disrupts organelle network homeostasis (47). Excess lipids and accumulated lipid droplets generate abundant intermediate metabolites, including acyl-CoA, diacylglycerol, ceramides, and acylcarnitines, which promote mitochondrial functional overload (48, 49). Persistent hyperglycemia, impaired fatty acid oxidation, and chronic inflammation synergize with lipotoxicity to amplify oxidative stress cascades (50). Metabolic substrate overload substantially impairs the mitochondrial electron transport chain (ETC), promoting excessive superoxide and mitochondrial reactive oxygen species (mtROS) production (20). Moreover, elevated arachidonic acid in PCOS follicular fluid and nervonic acid accumulation within granulosa cells act as direct lipotoxic mediators, facilitating substantial local mitochondrial oxidative stress. Ultimately, mitochondria-derived oxidative stress constitutes a critical pathological nexus linking glucolipid overload to tissue IR (30, 51, 52).

Sustained glucolipotoxicity promotes ROS overload and physically disrupts mitochondrial network dynamics. Cellular fuel overload aberrantly activates proinflammatory NF-κB signaling in target cells, thereby modulating mitochondrial dynamics (53). This dynamic s2t upregulates the fission protein DRP1 and significantly suppresses the fusion protein MFN2 (53, 54). This imbalance promotes pathological fragmentation. Microscopically, the healthy reticular structure is compromised, manifesting as increased aspect ratio and perimeter, organelle swelling, cristae depletion, and a threefold increase in mitochondrial fragmentation (53, 55, 56). Additionally, the synergistic effects of PCOS-specific hyperandrogenism and lipid dysmetabolism further exacerbate ROS accumulation and promote excessive mitochondrial fission (57, 58).

Glucolipotoxicity-induced structural damage contributes to ETC complex dysfunction and cellular metabolic arrest. Accumulated lipid metabolites, such as nervonic acid, attenuate the core respiratory chain proteins CYTB and NDUFB8, substantially impeding electron transfer (30). Prolonged glucolipotoxicity impairs mitochondrial respiration and oxidative phosphorylation by over 50%, alongside a substantial decrease in membrane potential and substantial mitochondrial DNA depletion (50, 53, 59). This substantial respiratory defect impedes the tricarboxylic acid cycle, substantially reducing ATP synthesis (60, 61). To mitigate the energy deficit, ovarian granulosa cells shift toward anaerobic glycolysis for metabolic compensation. However, this alternative pathway remains energetically insufficient. Ultimately, this global disruption of metabolic homeostasis deprives oocytes of indispensable metabolic support, establishing a core pathological mechanism that underlies PCOS follicular dysplasia (62, 63).

### 2.2 Collapse of protein homeostasis and chronic ER stress

The core pathogenesis of PCOS lies in intra- and extracellular proteostasis dysfunction, substantially promoting its complex metabolic and reproductive phenotypes. Proteome-wide analyses demonstrate that genetic variations in circulating proteins mediate PCOS onset, establishing proteostatic dyshomeostasis as a crucial pathogenic factor (22). Within the ovarian microenvironment, impaired stress granule clearance disrupts local protein network homeostasis, promoting toxic accumulation and early organelle dysfunction (64). Ectopic lipid accumulation in metabolic target organs suppresses major urinary protein 1 secretion, substantially disrupting cellular protein balance (65). Therefore, synergistic systemic and local protein network impairments form the primary pathological foundation for PCOS microenvironmental deterioration.

Proteostasis disruption significantly promotes ER stress within the PCOS microenvironment. Prolonged hyperandrogenism and hyperinsulinemia provoke substantial unfolded protein aggregation in the ER lumen, sustaining unfolded protein response (UPR) overactivation in ovarian granulosa cells (66). Experimental models suggest that androgen-mediated systemic metabolic dysregulation contributes to persistent ER stress and the upregulation of its sensor proteins (15, 67). In the classical UPR cascade, excess palmitic acid activates the IRE1 pathway and upregulates XBP1 to disrupt metabolic homeostasis, while simultaneously overactivating ATF4 downstream of the PERK/eIF2α/ATF4/CHOP signaling axis to promote ovarian cytotoxicity (17, 68, 69). Continuous metabolic stress progressively shifts the UPR from protective adaptation to a deleterious pro-apoptotic state. In contrast to acute adaptive responses, this chronic ER stress substantially facilitates the progression of severe IR (20, 70). Elevated PDIA4 chaperone expression promotes chronic adipose tissue inflammation, while prevalent hyperhomocysteinemia further enhances global metabolic toxicity via ER stress activation (71, 72).

Proteostasis dysfunction and chronic ER stress intertwine to generate a substantial synergistic toxic network in PCOS. Unfolded protein aggregation initially promotes persistent stress, whereas hyperactivated ER stress reciprocally impedes normal protein synthesis and secretion, establishing a vicious cycle of localized toxicity. Additionally, oxidative stress-triggered ER stress intimately couples with systemic oxidative pathways to jointly promote metabolic dysregulation (68, 73). Ultimately, the synergy between proteostatic dyshomeostasis and ER stress constitutes the core organelle pathology facilitating extensive oxidative damage, hyperinflammation, and profound reproductive endocrine dysfunction in granulosa cells (74).

Organelle dyshomeostasis operates through interconnected subcellular networks rather than isolated events. Persistent upstream stimuli, including hyperinsulinemia, hyperandrogenism, and saturated fatty acids such as palmitic acid, Promote reactive oxygen species (ROS) overload and DRP1-mediated mitochondrial fission (53, 57), while facilitating pathological luminal accumulation of unfolded proteins. This luminal overload chronically hyperactivates the PERK, IRE1, and ATF6 unfolded protein response (UPR) pathways (17, 66). Chronic ER stress acts as a pivotal intermediate to downregulate autophagy-related proteins such as ATG7 and suppress PINK1/PARKIN-mediated mitophagy via the IRE1-XBP1 axis (17, 54). Defective lysosomal acidification and autophagosome-lysosome fusion impair the clearance of damaged mitochondria and protein aggregates. Ultimately, these converging defects establish a self-reinforcing feedforward loop of mitochondrial injury, ER stress, and autophagic arrest, exacerbating intracellular metabolic toxicity (75, 76).

### 2.3 Lysosomal dysfunction and impaired autophagy

In the PCOS microenvironment, autophagic flux interruption plays a pivotal role in the pathogenesis of follicular developmental arrest and systemic dyshomeostasis (38). Substantial autophagic defects manifest in both circulating immune cells and ovarian follicles of PCOS patients (6, 75). Aberrant local autophagosome accumulation substantially obstructs macromolecular clearance (36). Hyperandrogenism substantially inhibits granulosa cell mTOR signaling, markedly initiating autophagy (50). Abnormal nucleolar protein BOP1 downregulation mediates nucleolar stress, activating aberrant autophagic pathways amidst disrupted homeostasis (77). This process relies on intricate regulatory networks, wherein AMPK and mTOR pathways modulate granulosa cell autophagy via ULK1 phosphorylation, synergized by circadian clock genes (78, 79). Crucially, ER stress-induced XBP1 overexpression downregulates ATG7 by promoting FOXO1 ubiquitination, substantially suppressing early autophagic initiation (17). A diminished LC3B-II/I ratio alongside substantial P62 substrate accumulation in granulosa cells indicates substantial deficits in autophagosome biogenesis and degradation (54, 80).

Alongside autophagosome accumulation, compromised lysosomal acidification and phagocytic capacity contribute to autophagosome-lysosome fusion impairment. Under metabolic stress, hypoxia-inducible factor 2α (HIF-2α) stimulates TFEB phosphorylation to downregulate core lysosomal genes, inducing lysosomal stress and attenuating immune cell phagocytosis (81). Additionally, V-ATPase-interacting protein NCOA7 deficiency promotes ectopic stress granule accumulation in ovarian cells, substantially impeding macromolecular degradation (64). PCOS-associated hyperandrogenemia aberrantly activates chaperone-mediated autophagy via LAMP2A upregulation, accelerating specific functional protein clearance and disrupting lysosomal homeostasis (82). Ultimately, the convergence of autophagosome accumulation and downstream degradative dysfunction exacerbates local microenvironmental dyshomeostasis.

Intertwined lysosomal dysfunction and autophagic arrest disrupt the mitophagy-lysosomal axis, restricting damaged mitochondria clearance and promoting aberrant metabolite accumulation (76). Colocalization analyses demonstrate substantially suppressed lysosomal degradation of damaged mitochondria in PCOS granulosa cells, accompanied by marked downregulation of core mitophagy proteins PINK1 and PARKIN (54, 83). Without the stress sensor HSP72, depolarized mitochondria fail to recruit PARKIN for target protein ubiquitination, thereby losing the critical prerequisite for autophagic degradation initiation (84). This defective clearance machinery not only diminishes LC3 and TOMM20 expression in ovarian and hepatic tissues but also promotes substantial accumulation of damaged mitochondrial networks, ultimately exacerbating apoptosis and tissue functional decline within the PCOS pathological microenvironment (40).

In summary, upstream metabolic triggers such as free fatty acids, hyperglycemia, and hyperandrogenism disrupt intracellular homeostasis in target cells by damaging the mitochondrial respiratory chain, sustaining unfolded protein response (UPR) activation in the ER, and blunting lysosomal autophagic flux (50, 66, 75). However, organellar stress extends beyond cytoplasmic boundaries. Upon breaching subcellular defenses, leaked mitochondrial reactive oxygen species (mtROS) and mitochondrial DNA (mtDNA) act as key endogenous danger signals to activate innate immune signaling pathways, including cGAS-STING, NF-κB, and HIF-1α (52, 53, 85). This shift from cellular stress to tissue-level immunity initiates a feedback loop that promotes pro-inflammatory M1 macrophage polarization and Warburg-type glycolytic reprogramming (61, 86). Ultimately, this self-amplifying cascade propagates localized subcellular injury into systemic low-grade chronic inflammation, substantial IR, and reproductive dysfunction (11).

## 3 Organelle-stress-mediated immunometabolic reprogramming in PCOS

Organelle dyshomeostasis translates localized metabolic stress into systemic pathogenic signaling. Mitochondrial reactive oxygen species (mtROS) leakage and aberrant mitochondria-associated membrane (MAMs) reorganization function as endogenous danger signals. Bridging subcellular damage with innate immunity, these intermediate mediators facilitate localized inflammatory responses. Ultimately, this cascade promotes macrophage immunometabolic reprogramming toward pro-inflammatory M1 and Warburg phenotypes (**Figure 2**).

**Figure 2:**
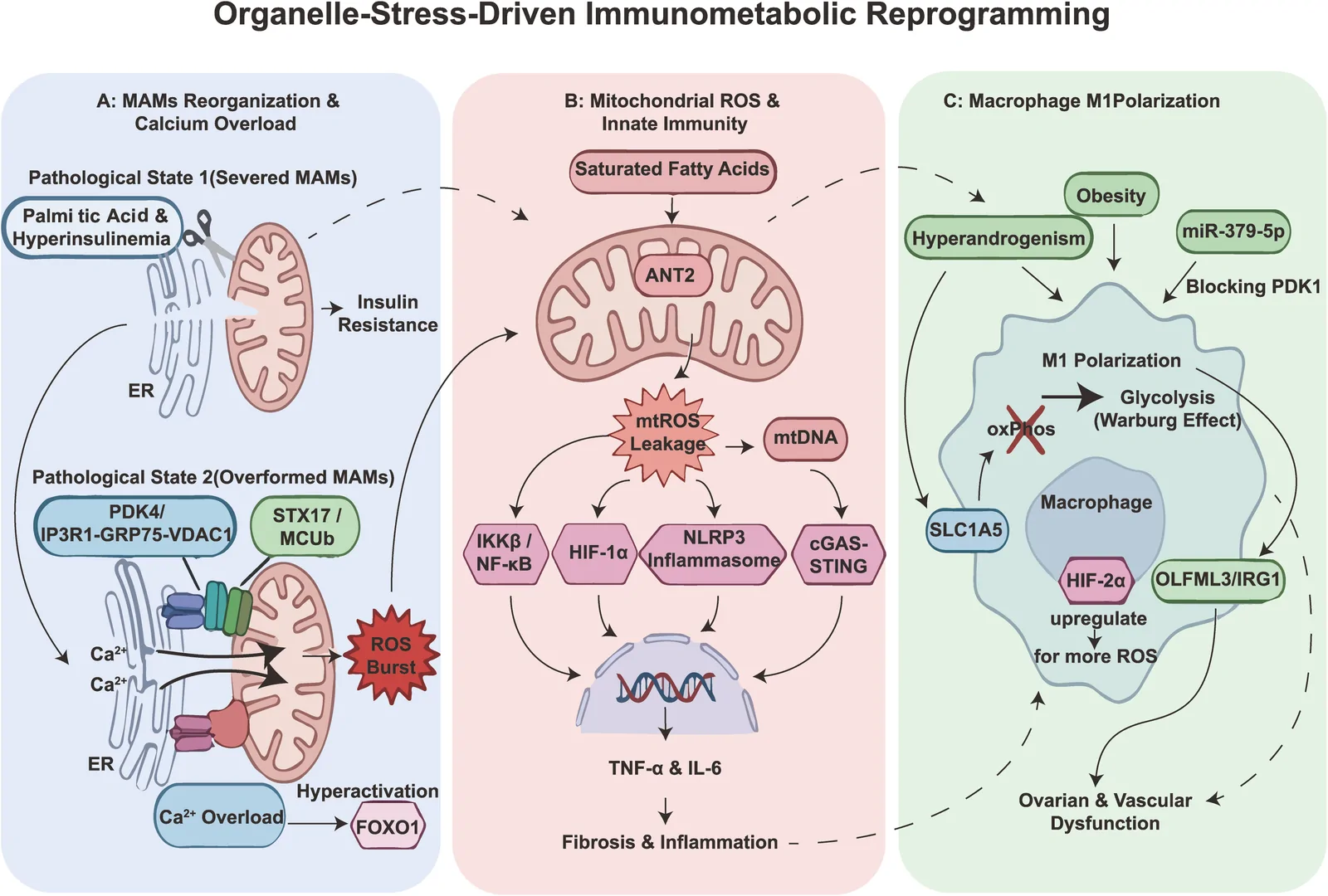
Mechanisms of organelle stress-mediated immunometabolic reprogramming. In PCOS, hyperinsulinemia, saturated fatty acids, hyperandrogenism, and obesity promote organelle dysfunction, thereby exacerbating IR, inflammation, and ovarian-endothelial damage. **(A)** MAMs reorganization and calcium overload. Hyperinsulinemia and palmitate disrupt MAMs to contribute to IR. Conversely, aberrant PDK4/IP3R1-GRP75-VDAC1 and STX17/MCUb interactions promote excessive formation of MAMs. Dysregulated ion channels promote substantial calcium overload, reactive oxygen species (ROS) accumulation, and substantial FOXO1 activation. **(B)** Mitochondrial ROS and innate immunity. Saturated fatty acids target ANT2, promoting mitochondrial ROS (mtROS) and mtDNA leakage. These messengers activate the IKKβ/NF-κB, HIF-1α, NLRP3, and cGAS-STING pathways, facilitating TNF-α and IL-6 transcription to contribute to fibrotic inflammation. **(C)** Macrophage M1 polarization. Hyperandrogenism, obesity, and miR-379-5p-mediated PDK1 inhibition promote macrophage polarization toward the M1 phenotype. This reprogramming impairs oxidative phosphorylation (oxPhos), enhancing glycolysis. HIF-2α amplifies ROS release, while the OLFML3/IRG1 axis accumulates itaconate. Ultimately, secreted cytokines and cytotoxic mediators exacerbate ovarian-endothelial dysfunction. MAMs, Mitochondria-Associated Endoplasmic Reticulum Membranes; ER, Endoplasmic Reticulum; PDK4, Pyruvate Dehydrogenase Kinase 4; IP3R1, Inositol 1, 4,5-Trisphosphate Receptor Type 1; GRP75, Glucose-Regulated Protein 75; VDAC1, Voltage-Dependent Anion Channel 1; STX17, Syntaxin 17; MCUb, Mitochondrial Calcium Uniporter dominant-negative beta subunit; ROS, Reactive Oxygen Species; FOXO1, Forkhead Box Protein O1; ANT2, Adenine Nucleotide Translocator 2; mtROS, Mitochondrial Reactive Oxygen Species; mtDNA, Mitochondrial DNA; IKKβ, Inhibitor of Nuclear Factor Kappa-B Kinase Subunit Beta; NF-κB, Nuclear Factor Kappa-light-chain-enhancer of Activated B Cells; HIF-1α, Hypoxia-Inducible Factor 1-alpha; NLRP3, NLR Family Pyrin Domain Containing 3; cGAS, Cyclic GMP-AMP Synthase; STING, Stimulator of Interferon Genes; TNF-α, Tumor Necrosis Factor-alpha;IL-6, Interleukin 6; PDK1, Pyruvate Dehydrogenase Kinase 1;oxPhos, Oxidative Phosphorylation;SLC1A5, Solute Carrier Family 1 Member 5; HIF-2α, Hypoxia-Inducible Factor 2-alpha; OLFML3, Olfactomedin-like 3; IRG1, Immune-Responsive Gene 1.

### 3.1 mtROS as a key second messenger in innate immune activation

Multi-omics analyses reveal that lipotoxic factors, notably free fatty acids, synergize with hormonal imbalances to construct a complex metabolic toxic microenvironment in PCOS (69). Under this metabolic and hyperandrogenic stress, nutrient overload selectively targets the mitochondrial network within ovarian granulosa cells and surrounding tissues. ANT2 mediates lipid-induced mitochondrial permeability transition, promoting substantial mitochondrial reactive oxygen species (mtROS) generation and cytosolic escape (87). Importantly, cytosolic mtROS extends beyond its role as a mere oxidative byproduct, functioning as a pivotal redox second messenger that regulates specific gene expression and modulates cellular metabolic adaptation (28). mtROS transitions from a damage mediator to a core signaling hub, directly linking initial organelle injury to systemic chronic inflammation in PCOS (29).

As an upstream mediator of innate immunity, cytosolic mtROS substantially activates oxidative stress-sensitive pathways. Sustained metabolic overload and mitochondrial dyshomeostasis persistently activate the core intracellular IKKβ-NF-κB cascade (53). The hyperandrogenic PCOS milieu facilitates substantial crosstalk between androgen signaling and NF-κB, amplifying local ovarian inflammation and contributing to endothelial dysfunction (13, 88). Additionally, pathologically upregulated MiR-93-5p synergistically activates classical NF-κB signaling to exacerbate ovarian inflammatory responses (89). In the context of persistent mtROS and related stress signals, PCOS monocytes exhibit substantial depletion of endogenous inhibitors alongside aberrant p65 enrichment. This indicates a constitutively primed innate immune state associated with organelle stress, persisting even under fasting baseline conditions (18). mtROS also crosstalks with HIF-1α, a crucial genetic susceptibility node in PCOS, to cooperatively facilitate downstream cell death and immunometabolic reprogramming (22).

Following the sustained activation and nuclear translocation of transcription factors like NF-κB and HIF-1α by mtROS, robust transcription of downstream pro-inflammatory genes is initiated across multiple target tissues. The mRNA expression and release of core pro-inflammatory mediators, including interleukin-6 (IL-6), tumor necrosis factor-α (TNF-α), and interferon-γ (IFN-γ), increase substantially; a phenomenon well-documented in androgen-exposed uterine models and PCOS placental tissues (14, 90). This mtROS-initiated signaling cascade not only promotes marked TNF-α secretion from ovarian granulosa cells to disrupt follicular immune homeostasis and contribute to concurrent renal injury, but also propagates through the circulation to establish a systemic pro-inflammatory microenvironment in distal organs such as the heart, liver, and adipose tissue (47, 91, 92). Ultimately, this mitochondria-mediated, pan-tissue inflammatory cascade establishes a persistent network of immunometabolic dysregulation and chronic low-grade inflammation, substantially impairing both reproductive and systemic physiological functions in PCOS patients (11, 93).

### 3.2 Oxidative stress modulates macrophage metabolic reprogramming

In the PCOS pathological network, persistent oxidative stress and chronic low-grade inflammation forge the core microenvironment facilitating disease progression (45). As central innate immune regulators, macrophages link local tissue stress to systemic inflammation through phenotypic polarization and metabolic reprogramming (3). The hyperandrogenic milieu specifically disrupts immune homeostasis by activating androgen receptors, modulating macrophage remodeling and polarization within uterine, ovarian, and visceral adipose tissues (12, 14). Dysfunctional adipose tissue amplifies systemic inflammatory cascades via adipokine modulation and androgen crosstalk (39, 94). Therefore, excessive macrophage infiltration into the PCOS ovary constitutes the primary pathological event promoting local immunoinflammatory dyshomeostasis (3, 11).

The oxidative microenvironment substantially promotes macrophage polarization toward the pro-inflammatory M1 phenotype. Granulosa cell-derived miR-379-5p facilitates M1 polarization by repressing PDK1, concurrently antagonizing tissue-reparative M2 polarization. The elevated local M1/M2 ratio substantially aggravates the ovarian immune microenvironment (86). Although compensatory mechanisms, notably heme oxygenase-1 upregulation, attempt to mitigate inflammation via M2-like remodeling (80, 95), substantial oxidative and lipotoxic stress ultimately overwhelms this adaptive balance. Furthermore, defective mitophagy in peripheral blood mononuclear cells contributes to intracellular oxidative stress accumulation, substantially amplifying pro-inflammatory immune polarization (75).

Metabolically, M1 polarization within the PCOS toxic microenvironment entails substantial cellular rewiring, characterized by a marked shift from oxidative phosphorylation to glycolysis. Hyperandrogenism further amplifies this metabolic transition. Both macrophages and ovarian granulosa cells exhibit a tumor-like Warburg effect, synergistically exacerbating the local oxidative stress burden (61). Under PCOS-associated obesity and lipotoxicity, adenine nucleotide translocase 2 (ANT2) emerges as a critical metabolic node mediating adipose tissue macrophage activation (87). Similarly, in distal comorbidities like non-alcoholic fatty liver disease, hypoxia-inducible factors upregulate ANT2 to facilitate mitochondrial permeability transition, promoting substantial mitochondrial ROS production and pro-inflammatory polarization of recruited macrophages (81). Furthermore, metabolic intermediates critically regulate PCOS immunity. OLFML3 orchestrates mitochondrial localization of IRG1 to modulate macrophage mitochondrial function, elucidating local immunometabolic reprogramming mechanisms (96). Metabolite-associated innate immune responses in PCOS display substantial systemic sensitivity; even a single postprandial nutrient challenge rapidly promotes marked intense pro-inflammatory receptor expression in peripheral leukocytes, underscoring rapid immune axis activation (97). Ultimately, within the hyperinsulinemic and hyperandrogenic pathological network, glycolytically reprogrammed macrophages secrete abundant pro-inflammatory cytokines, including TNF-α and IL-6. This signaling cascade substantially disrupts local ovarian steroidogenesis to aggravate ovulatory dysfunction, while circulating macrophages extensively infiltrate distal target organs, facilitating PCOS-specific systemic cardiovascular inflammation and ischemic tissue injury (3, 98).

The pathogenesis of PCOS underscores the fundamental sex-specificity of immune pathways, where hyperandrogenism collapses the female immuno-endocrine microenvironment and disrupts estrogen-mediated homeostasis through elevated T-cell interferon-gamma (IFN-γ) secretion (99). This sex-specific immune remodeling is markedly amplified within female reproductive target organs, where local androgen excess drives immune cell dysregulation—including natural killer cell proliferation, monocyte-macrophage polarization, and macrophage pyroptosis—which suppresses local estrogenic protection and dictates ovarian pathological progression (14, 99, 100).

### 3.3 Abnormal reorganization of the mitochondrial-ER cristae

In PCOS, the structural remodeling of MAMs critically disrupts inter-organelle communication and systemic homeostasis. This physical interface substantially regulates cellular energy metabolism, organelle quality control, and insulin signaling (101). However, metabolic stress from hyperinsulinemia and lipotoxicity promotes substantial MAM distortion. It physically distorts hepatic MAMs, thereby disrupting endogenous cellular calcium homeostasis (70). Palmitic acid-induced lipid overload substantially disrupts MAM physical contacts. This reorganization represents a core mechanism contributing to target organ IR (48).

Conversely, metabolic disorders frequently promote pathological MAM overformation, a structural aberration highly relevant to the metabolic complications of PCOS. Although direct microstructural evaluations of cardiac and skeletal muscle in specific PCOS models remain scarce, shared pathologies with generalized obesity models provide critical mechanistic insights. Upregulated skeletal muscle PDK4 interacts with the IP3R1-GRP75-VDAC1 complex to abnormally stabilize mitochondria-ER contacts (101, 102). Overexpressed syntaxin 17 (STX17) facilitates pathological MAM formation in cardiomyocytes (109). Crucially, the structural and physiological integrity of these inter-organelle junctions is intrinsically dictated by the localized metabolic hub mTOR (103). Given the substantial lipotoxic burden and IR inherent to PCOS, these specific organelle-level aberrations, notably the PDK4-mediated MAM structural distortions, represent conserved pathogenic mechanisms facilitating PCOS-associated myopathy and cardiovascular risk.

This structural distortion substantially disrupts the intracellular calcium signaling network. Excessive MAM formation establishes uncontrolled ER-to-mitochondria ion channels, promoting substantial mitochondrial calcium overload and functional impairment (102). Mechanistically, STX17-mediated calcium influx depends on the abnormal interaction between its Habc domain and the dominant-negative β-subunit of the mitochondrial calcium uniporter (MCUb) (104). Additionally, pathological HB-EGF signaling synergistically exacerbates intracellular calcium accumulation, substantially activating damage-responsive transcription factors including FOXO1 (59). Under substantial metabolic stress, this dysregulated calcium transfer promotes damaged mitochondria to release substantial ROS, reinforcing a vicious mitochondria-ER communication loop (105).

This dysfunctional physical coupling extends beyond cellular oxidative stress to disrupt systemic metabolic homeostasis. Subcellular MAM structural integrity plays a pivotal role in hepatic insulin sensitivity, with its imbalance facilitating substantial organ-level IR (106, 107). Therefore, aberrant MAM communication bridges structural organelle defects with distal metabolic stress, systemic tissue degeneration, and neuroendocrine dysregulation (105).This dysregulation extends to the nucleus. Altered mitochondrial DNA epigenetic modifications substantially impair mitochondria-nucleus signaling in PCOS, exacerbating cellular energy dysfunction (108). Simultaneously, systemic trace element imbalances, notably elevated serum copper, combined with declined total antioxidant capacity, entirely dismantle baseline defenses against oxidative stress (109). Reduced microbiota-derived metabolites like indole-3-propionic acid (IPA) impede the AhR-NLRP3 signaling axis, preventing the resolution of organelle damage-induced systemic inflammation. Ultimately, these multidimensional metabolic toxicities progressively contribute to the deterioration of systemic PCOS pathology (110).

Ultimately, structural reorganization of MAMs represents a pivotal convergent node in the pathological landscape of PCOS. Persistent upstream metabolic insults, including lipotoxicity, hyperglycemia, and hyperandrogenism (15, 47), disrupt MAM tethering by promoting aberrant expression of key proteins such as PDK4 and STX17 (102, 104). Pathological MAM remodeling facilitates substantial ER-to-mitochondrial Ca2+ transfer, contributing to mitochondrial calcium overload and a secondary increase of mitochondrial reactive oxygen species (mtROS) (103, 104). In turn, elevated mtROS and downstream pro-inflammatory cytokines such as TNF-α and IL-6 further exacerbate ER stress while sustaining M1 and Warburg-type metabolic reprogramming in macrophages. This crosstalk establishes a self-amplifying feedback loop comprising ER stress, MAM reorganization, calcium overload, and immunometabolic polarization (3, 53, 70). Ultimately, this self-sustaining microenvironmental disturbance perpetuates widespread granulosa cell atresia, follicular arrest, and multi-organ systemic IR (20, 111, 112).

## 4 Oxidation-mediated programmed cell death networks

Macrophage immunometabolic reprogramming contributes to persistent oxidative stress and chronic inflammation within the PCOS microenvironment. This oxidative-inflammatory network impairs cellular antioxidant defenses. Functioning as a critical feedback amplification effect, programmed cell death further contributes to this pathogenic cascade. The pathological milieu facilitates lipid peroxidation-dependent ferroptosis, inflammasome-dependent pyroptosis, alongside dysregulated apoptosis and autophagy in granulosa cells and macrophages. Released damage-associated molecular patterns (DAMPs) and exosomes propagate this localized injury into systemic dysfunction.

### 4.1 Ferroptosis in PCOS

In PCOS, ferroptosis constitutes a novel lipid peroxidation-dependent programmed cell death pathway, mediated primarily by iron overload and lipid peroxidation, that negatively impacts ovarian granulosa cells and alters the reproductive microenvironment (37, 113). Persistent reactive oxygen species accumulation mediates structural damage in ovarian lesions (114). This redox imbalance-dependent pathway impairs oocyte quality and *in vitro* fertilization outcomes (115), highlighting lipid peroxidation as a key component of PCOS pathogenesis (116). This dysregulated ferroptosis network propagates systemically, acting as a pathological hub linking multidimensional endocrine dysregulation to multi-organ dysfunction (117).

The propagation of lipid peroxidation stems from the impairment of cellular antioxidant defenses, primarily the GPX4 and SLC7A11 transporters. GPX4, the primary enzyme neutralizing lipid peroxides, is downregulated in PCOS granulosa cells and ovarian tissues (118). Epigenetically, methyl-binding domain 2 (MBD2) represses GPX4 transcription, facilitating granulosa cell ferroptosis (119). The antioxidant substrate transporter SLC7A11 is broadly inhibited. Molecularly, serum exosomal miR-128-3p deficiency permits cross-tissue CSF1 upregulation in granulosa cells (120). This cascade activates the p38 and JNK pathways, which suppresses NRF2-mediated SLC7A11 transcription, compromising the SLC7A11 antioxidant system and leading to severe ferroptosis and glutathione depletion (120). This antioxidant deficiency-triggered ferroptosis propagates distally, contributing to endometrial epithelial fibrosis (121). Although the endogenous stress regulator SESN2 attempts to mitigate ferroptosis via maintaining specific protein lactylation, notably PRDX6 lactylation (122), these compensatory mechanisms remain insufficient against systemic multidimensional lipotoxicity.

Alongside antioxidant compromise, disrupted intracellular iron homeostasis sensitizes cells to ferroptosis. The PCOS hyperandrogenic microenvironment exacerbates reproductive iron metabolism disorders (55). It accelerates chaperone-mediated autophagy in trophoblasts, promoting the degradation of the iron storage protein FTH1 (82). This FTH1 downregulation facilitates intracellular free iron accumulation, triggering trophoblast ferroptosis and elevating early miscarriage risk. Therapeutically, agents like metformin can competitively inhibit androgen binding to prevent this FTH1 degradation, thereby rescuing trophoblasts from ferroptosis (82, 123). Dehydroepiandrosterone-induced hormonal dysregulation disrupts endometrial stromal cells, with dipeptidyl peptidase 4 acting as a central node governing endometrial ferroptosis tolerance (37). Aberrant microRNAs, notably miR-93-5p, synergistically activate NF-κB signaling to amplify granulosa cell apoptosis and ferroptosis (89). Ultimately, suppression of the core metabolic deacetylase SIRT1 further limits endogenous ferroptosis defenses (124). Conversely, activated SIRT1 deacetylates transcription factors to upregulate antioxidant genes and promote mitochondrial biogenesis, which substantially inhibits ferroptosis (33, 124, 125). Targeted therapeutic strategies also intervene in these pathways: specific ferroptosis inhibitors, effectively Fer-1, precisely disrupt these lipid peroxidation networks (55, 126). while rosastat selectively targets the ferroptosis axis (22). Additionally, agents like phycocyanin attenuate ferroptosis via NRF2-mediated pathways (126, 127), and specific plant derivatives modulate these intricate ferroptosis networks (118, 128, 129).

Ferroptosis regulation within the reproductive microenvironment exhibits pronounced sex-specific characteristics driven by sex hormone dynamics. In hyperandrogenic disorders such as PCOS, diminished estrogenic protection synergizes with androgen excess to exacerbate reproductive cell damage by elevating reactive oxygen species (ROS) and downregulating glutathione peroxidase 4 (GPX4), creating a pathogenic positive-feedback loop that further aggravates hyperandrogenemia (82, 113, 121, 130). Current paradigms highlight androgenic promotion, loss of estrogenic shielding, and epigenetic iron dysregulation as central drivers, leaving deep genomic determinants like X-chromosome-linked protection to be further elucidated.

### 4.2 Pyroptosis in PCOS

In PCOS, pyroptosis serves as a notable inflammatory programmed cell death pathway contributing to reproductive microenvironment degeneration and systemic metabolic disorders. Sustained androgen exposure, gut microbiota dysbiosis-derived macrophage polarization, and elevated metabolic factors, notably phosphatidylinositol, facilitate target tissue pyroptosis (99, 131). This process substantially relies on upstream organelle stress networks, particularly the feedback loop intertwining ER stress and mitochondrial dysfunction. Local lipotoxicity, notably abnormal nervonic acid accumulation, substantially exacerbates ER stress to promote marked mitochondrial oxidative stress (30, 132). Substantially produced and leaked mtROS act as critical upstream mediators. These species markedly activate the dormant cytosolic NLRP3 inflammasome, transforming organelle damage into a downstream inflammatory cascade.

Under the synergistic influence of mtROS and hyperandrogenism, NLRP3 readily activates, contributing to multiprotein complex assembly (7, 133). Activated NLRP3 recruits the apoptosis-associated speck-like protein containing a CARD (ASC) and procaspase-1 to form the core pyroptotic cleavage engine. Clinically, peripheral blood mononuclear cells from PCOS patients exhibit significantly elevated expression of key inflammasome components, namely NLRP3, ASC, and procaspase-1. This systemic elevation indicates the substantial polarization, initiation, and pre-activation of the innate immune inflammatory network (134). The assembled complex then catalyzes procaspase-1 auto-cleavage, generating active caspase-1 and mediating the molecular gateway to inflammatory cell death (127).

Active caspase-1 precisely transmits the death signal to Gasdermin D (GSDMD). Specific cleavage relieves GSDMD auto-inhibition, prompting its free N-terminal domain to oligomerize and form extensive cell membrane pores (127, 131). These GSDMD pores disrupt physical cellular integrity, mediating osmotic swelling and lytic pyroptosis in granulocytes and macrophages, while simultaneously acting as channels for pro-inflammatory cytokine release. Caspase-1 mediates the maturation cleavage of pro-inflammatory cytokines, facilitating the substantial extracellular release of active IL-1β and IL-18 through these pores (135). Within the ovary, these mediators promote a chronic low-grade inflammatory response that disrupts steroidogenesis. This constitutes the core mechanism contributing to follicular hypoplasia, ovulatory dysfunction, and progressive ovarian fibrosis (15, 30). This NLRP3- and GSDMD-mediated inflammatory cascade extends beyond the reproductive system to distal metabolic targets, notably the myocardium. This widespread propagation contributes to cellular structural damage and systemic metabolic syndrome (136, 137), ultimately confirming the pivotal role of the peroxidation-pyroptosis network in multidimensional PCOS pathophysiology.

### 4.3 Apoptosis and autophagy in PCOS

In PCOS, extensive granulosa cell apoptosis promotes significant ovarian dysfunction and anovulation (138). Oxidative stress facilitates this process, damaging organelles and epigenetically modulating apoptotic genes via chromatin remodeling (139). Excessive ROS and persistent ER stress synergistically trigger irreversible granulosa cell apoptosis (68, 74). Molecularly, substantial ROS accumulation weakens the interaction between the E3 ubiquitin ligase WWP2 and pro-apoptotic BAK. This impairs BAK ubiquitination, culminating in a marked pro-apoptotic signal accumulation (138). The nuclear receptor NR1D1 suppresses HSD17B12 transcription, amplifying ROS-induced apoptosis (139). Ultimately, a substantial pro-apoptotic BAX to anti-apoptotic BCL-2 imbalance activates Caspase-3, contributing to cell death. This apoptotic cascade extends centrally; hyperandrogenism stimulates microglial pro-inflammatory cytokine release, thereby exacerbating hypothalamic neuroendocrine damage (140).

Normal follicular development and atresia rely on a delicate physiological balance between apoptosis and autophagy (139). Initially, mild metabolic stress induces protective autophagy to clear damaged mitochondria and misfolded proteins, buffering organelle stress. However, the PCOS pathological microenvironment substantially disrupts this balance, altering the autophagic network’s function. Extensive evidence reveals marked autophagic flux interruptions and defective mitophagy across PCOS follicular stages and circulating immune cells (6, 75). Crucially, granulosa cell co-localization confirms the substantially impeded degradation of damaged mitochondria within the autophagic pathway (83). This dysregulated flux widely disrupts systemic metabolism and constitutes a core pathological mechanism contributing to infertility and reproductive decline (38). Ultimately, this ineffective autophagy, defined by elevated initiation combined with substantial stalling, promotes persistent intracellular waste accumulation. Rather than rescuing damaged granulosa cells, it synergizes with the progressive apoptotic network to facilitate delayed follicular development, excessive atresia, and widespread microenvironmental dysfunction (79).

### 4.4 Organelle-derived DAMPs and exosome-mediated amplification of systemic inflammatory cascades

In PCOS, local programmed cell death within ovarian granulosa and adipose tissues acts not as an endpoint, but as a critical trigger for systemic metabolic collapse. Extensive cellular barrier disruption, associated with severe peroxidation and pyroptosis, contributes to the systemic release of organelle-derived damage-associated molecular patterns (DAMPs). Lethal mitochondrial dysfunction releases elevated copies of free mitochondrial DNA (mtDNA) and oxidized DNA fragments, acting as potent endogenous stimulants that broadly amplify circulatory immune responses (77). Pyroptosis-mediated inflammasome activation via PKR promotes the substantial systemic release of the nucleocytoplasmic alarm protein HMGB1 (136). Locally released inflammatory cytokines, notably TNF-α, and intestinal barrier-derived endotoxins function collectively as systemic mediators. These efficiently transmit reproductive-immune imbalances to distal organs (141). This boundary-transcending inflammatory storm induces severe distal renal tubular cell apoptosis and dysfunction (91, 142), while concurrently propagating systemic oxidative stress across vascular endothelial cells to promote cardiovascular pathogenesis (143, 144).

Within this infectious inflammatory network, stable lipid-bilayer extracellular vesicles, primarily exosomes, sustainably transmit local metabolic toxicity and aberrant epigenetic signals. Pre-ovulatory follicular fluid and systemic plasma exosomes encapsulate substantial abnormal non-coding microRNAs, profoundly disrupting microenvironmental intercellular signaling (145, 146). Exosomal miRNA abundance directly correlates with impaired follicular steroidogenesis and diminished embryo quality (147). Molecularly, serum exosomal miR-128-3p deficiency induces severe ferroptosis (120). Differentially expressed plasma miR-126-3p and miR-146a-5p, alongside circadian-disrupted exosomes, substantially amplify local metabolic stress into targeted systemic damage (41, 123). Additionally, specific exosomes from preantral follicular granulosa or mesenchymal stem cells carrying miR-143-3p and miR-379-5p profoundly impede glycolysis and promote pro-inflammatory macrophage polarization (63, 86). Finally, gut microbiota-derived nanoscale EVs analogously traverse physiological barriers to distally regulate host neuroendocrine networks and systemic inflammation (148). In this context, diet plays a pivotal role in modulating gut microbiota composition, where diet-induced dysbiosis releases gut microbiota-derived extracellular vesicles and metabolites into circulation (110, 148). These circulating signals traverse the intestinal barrier to trigger low-grade systemic inflammation and oxidative stress in distal tissues. Targeting this gut-immune interface with functional food components—such as Dendrobium officinale oligosaccharides—has been shown to effectively mitigate systemic inflammatory responses by restoring microbial homeostasis (149), highlighting the potential of gut-targeted approaches in suppressing systemic inflammatory cascades.

The synergistic integration of substantial DAMP release and targeted exosome delivery fosters a systemic pathological cascade. This circulatory immune transformation exponentially increases monocyte-endothelial interactions, facilitating the transition from local metabolic inflammation into systemic ischemic cardiac injury and heightened cardiovascular risk (75, 98). The integration of damaged mitochondrial exosomes and inflammatory cytokines perpetuates a vicious oxidative stress-inflammation cycle (28, 29). Clinical evidence indicates that this multidimensional inflammatory storm predisposes PCOS patients to severe cardiovascular and metabolic syndromes (150, 151), while serving as a critical node facilitating distal osteoporosis (152). Persistent peripheral toxicity severely disrupts the uterine microenvironment, exacerbating high-fat diet-associated placental inflammation and endometrial dysfunction (31). Despite endogenous compensatory attempts by specialized pro-resolving mediators to restore vascular homeostasis (8), alongside buffering efforts from gut microbiota modulators and nuclear-mitochondrial epigenetic communications (25, 108), these physiological defenses can be overwhelmed by this amplified androgen-immune imbalance. This underscores PCOS as a widespread, multi-tissue metabolic disorder (12, 153).

## 5 Oxidative-inflammatory network-mediated multidimensional PCOS comorbidity

Localized microenvironmental collapse mediated by organelle damage and programmed cell death promotes systemic metabolic dysfunction in PCOS. Systemic dissemination of damage-associated molecular patterns (DAMPs) and exosomes carrying aberrant epigenetic signals substantially extends this pathology beyond the reproductive axis. Accordingly, multidimensional systemic complications emerge as the definitive downstream pathological outcomes of PCOS. The oxidative-inflammatory network propagates metabolic toxicity to distal organs, exacerbating severe glucolipid dysmetabolism, hepatic steatosis and fibrosis, alongside high-risk cardiovascular comorbidities (**Figure 3**).

**Figure 3:**
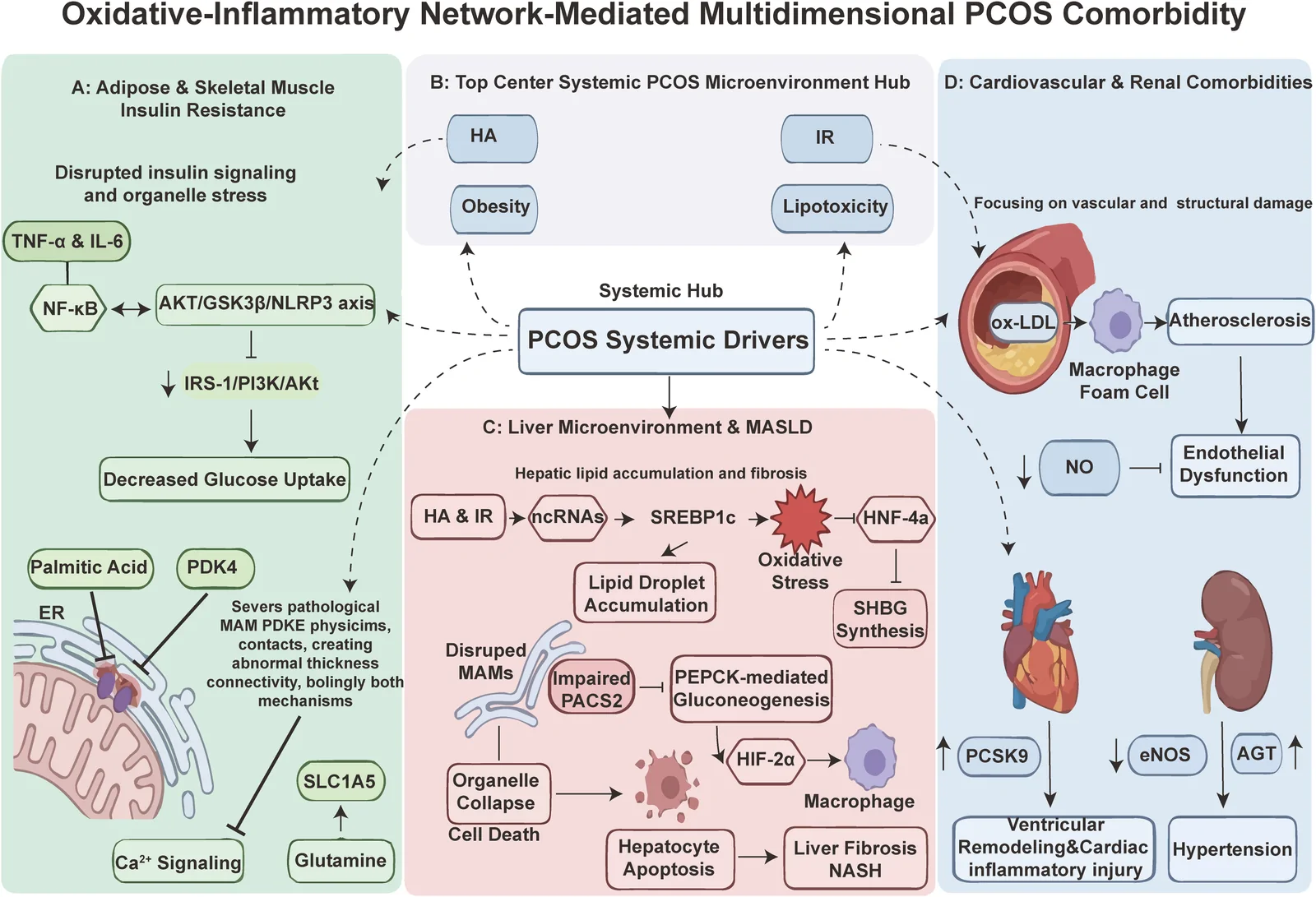
Oxidative-inflammatory networks mediate multidimensional PCOS comorbidities. **(A)** Musculoadipose IR. Inflammatory TNF-α/IL-6/NF-κB and AKT/GSK3β/NLRP3 cascades strictly impair IRS-1/PI3K/Akt signaling, reducing glucose uptake. Subcellularly, palmitate physically severs MAMs, whereas PDK4 pathologically hyperforms them; collectively, these structural aberrations disrupt critical signal transduction. Saturated fatty acids (SFA) upregulate SLC1A5-mediated glutamine uptake. **(B)** Key PCOS contributors. Hyperandrogenism (HA), obesity, IR, and lipotoxicity constitute the primary pathogenic factors. **(C)** Hepatic MASLD. Systemic factors promote SREBP1c activation, contributing to lipotoxicity and oxidative stress, whilst downregulating HNF-4a to suppress sex hormone-binding globulin (SHBG) synthesis. Subcellular organelle collapse accelerates PEPCK-mediated gluconeogenesis and hepatocyte apoptosis. Ultimately, HIF-2α-activated macrophages precipitate hepatic fibrosis and non-alcoholic steatohepatitis (NASH). **(D)** Cardiorenal comorbidities. Elevated oxidized low-density lipoprotein (ox-LDL) induces atherosclerosis, whereas decreased nitric oxide (NO) alters the ER secretome to promote endothelial dysfunction. Renally, HA hyperactivates the renin-angiotensin system, downregulating eNOS and driving hypertension. Cardiologically, upregulated PCSK9 and proinflammatory cytokines robustly amplify myocardial injury and ventricular remodeling. HA, Hyperandrogenism; IR, Insulin Resistance; TNF-α, Tumor Necrosis Factor-alpha; IL-6, Interleukin-6; NF-κB, Nuclear Factor kappa-B; AKT, AKT serine/threonine kinase; GSK3β, Glycogen Synthase Kinase 3 beta; NLRP3, NLR Family Pyrin Domain Containing 3; IRS-1, Insulin Receptor Substrate 1; PI3K, Phosphoinositide 3-kinase; PDK4, Pyruvate Dehydrogenase Kinase 4; ER, Endoplasmic Reticulum; MAMs, Mitochondria-Associated Endoplasmic Reticulum Membranes; Ca2+, Calcium ions; SLC1A5, Solute Carrier Family 1 Member 5; ncRNAs, Non-Coding RNAs; SREBP1c, Sterol Regulatory Element-Binding Protein 1c; HNF-4a, Hepatocyte Nuclear Factor-4a; SHBG, Sex Hormone-Binding Globulin; PACS2, Phosphofurin Acidic Cluster Disseminator 2; PEPCK, Phosphoenolpyruvate Carboxykinase; HIF-2α, Hypoxia-Inducible Factor 2-alpha; ox-LDL, Oxidized Low-Density Lipoprotein;NO, Nitric Oxide; PCSK9, Proprotein Convertase Subtilisin/Kexin Type 9; eNOS, Endothelial Nitric Oxide Synthase; AGT, Angiotensinogen; PCOS, Polycystic Ovary Syndrome; MASLD, Metabolic Dysfunction-Associated Steatotic Liver Disease; NASH, Non-Alcoholic Steatohepatitis.

### 5.1 PCOS and comorbidities in glucose and lipid metabolism

PCOS integrates reproductive dysfunction and hyperandrogenism with multidimensional metabolic disorders. Hyperandrogenism exacerbates systemic metabolic reprogramming by activating the glutamine transporter SLC1A5 to reshape amino acid metabolism (154). This progression is mediated by critical intermediate phenotypes, including systemic IR, impaired glucose tolerance, and hyperinsulinemia. Subcellularly, these conditions disrupt the physical and functional integrity of MAM, thereby impairing intracellular calcium homeostasis (102). Furthermore, hyperinsulinemia and ER stress collectively stimulate excessive hepatic lipid synthesis, disseminating lipotoxic substrates to distal tissues (70).

These metabolic shifts are severely aggravated by obesity, lipotoxicity, and persistent inflammation. Obesity-associated chronic inflammatory cascades significantly elevate cardiovascular risk (50). Moreover, palmitic acid exposure during PCOS-associated lipotoxicity severs MAM physical contacts, substantially disrupting calcium signaling and inducing IR (48).

This pathological progression is quantifiable through elevated HOMA-IR, fasting plasma glucose (FPG), and fasting insulin (73, 155, 156). Additionally, elevated serum uric acid (UA) signifies the coupling of metabolic and inflammatory networks (157). Meanwhile, accumulated pro-inflammatory cytokines, primarily TNF-α and IL-6, remodel immunometabolism to impair adipokine synthesis and disrupt post-receptor insulin signaling (158, 159). Conversely, protective gut microbial metabolites, notably IPA, are significantly depleted in PCOS (110).

Stress-activated NF-κB signaling converges with the AKT/GSK3β/NLRP3 axis to amplify inflammation in cardiac and visceral adipose tissues (137). This persistent inflammatory cascade directly suppresses the canonical IRS-1/PI3K/Akt pathway, impairing glucose uptake in skeletal muscle and adipose tissues to promote peripheral IR (160). Crucially, IRS-1/PI3K/Akt signaling impairment serves as a core molecular mediator intrinsic to PCOS pathophysiology, where concurrent hyperandrogenism directly synergizes with inflammatory stress to exacerbate this pathway blockade. Ultimately, this metabolic imbalance contributes to multi-organ dysfunction, presenting clinically as weight gain and hepatic ferroptosis (117). This pathological trajectory markedly elevates susceptibility to major comorbidities, including type 2 diabetes, coronary heart disease, and metabolic syndrome (73, 155, 156).

Targeted interventions offer promising therapeutic avenues to attenuate this comorbidity progression. For instance, IPA supplementation enhances insulin sensitivity and restores ovulatory cycles via the AhR-NLRP3 axis (110). Green tea extract demonstrates clinical efficacy in ameliorating metabolic dysregulation by promoting weight loss and normalizing glucose-insulin dynamics (156). Overall, strategies targeting signal transduction, adipokine regulation, and organelle physical coupling present a robust foundation for mitigating PCOS-associated glucolipid comorbidities and developing novel therapeutics (**Table 1**).

**Table 1:** Targeted therapeutic interventions and molecular mechanisms in PCOS and associated complications.

Category	Intervention	Target	Pathway	Molecular & cellular effects	Evidence level/stage	Clinical outcomes	Reference
Traditional Formulas	Yangjing Zhongyu Tang	Multi-organ ferroptosis	Ferroptosis inhibition	Suppresses multi-organ ferroptosis	Approved/Clinical Guideline	Alleviates PCOS phenotypes	(117)
	Qi Gong Wan	Adipose tissue and metabolic stress	NRF2/HO-1/Cyp1b1	Attenuates adipocyte hypertrophy and oxidative stress to reduce inflammation	Approved/Clinical Guideline	Ameliorates metabolic and reproductive dysfunction	(200)
	Bu-Shen-Tian-Jing Formula	Oxidative stress and glucose metabolism	Mitochondrial SIRT3 signaling	Restores mitochondrial function and regulates oxidative stress and glucose dysmetabolism	Approved/Clinical Guideline	Alleviates PCOS pathogenesis	(201)
Specific Bioactive Phytochemicals	Quercetin	Oocyte mitochondria	Mitochondrial ROS scavenging and anti-apoptosis	Scavenges mitochondrial ROS to reduce apoptosis and preserve spindle morphology	Preclinical (*In vitro*)	Promotes *in vitro* maturation and early embryonic development	(202)
	Curcumin and aerobic exercise	ER stress	Hyperandrogenism-induced IRE1α/XBP1	Suppressing hyperandrogenism-induced ER stress. This curtails reactive oxygen species, restores mitochondrial potential, and abrogates granulosa cell apoptosis.	Preclinical (*In vivo*) and Preclinical (*In vitro*)	Ameliorates follicular dysfunction	(203)
	Curcumin and metformin	IR and oxidative stress	NRF2 and antioxidant interaction networks	Enhances insulin sensitivity and inhibits oxidative damage	Metformin: Approved/Clinical Guideline •	Ameliorates reproductive and metabolic disorders	(204)
	Platycodin D	CD44 and granulosa cell ferroptosis	CD44-mediated ferroptosis inhibition	Upregulates CD44 to restore SLC7A11/GPX4,attenuating ferroptosis and oxidative damage	Preclinical (*In vivo*) and Preclinical (*In vitro*)	Ameliorates ovarian injury and follicular maldevelopment	(118)
	Leonurine	Granulosa cell ferroptosis and gut microbiota	SLC7A11/GPX4 axis and gut microbiota crosstalk	Activates SLC7A11/GPX4 to inhibit ferroptosis and regulate gut microbiota	Preclinical (*In vivo*) and Preclinical (*In vitro*)	Improves ovarian function, hormonal balance, and IR	(129)
	Marjoram	Insulin sensitivity and hormonal balance	Inflammation and oxidative stress blockade	Suppresses inflammation and oxidative damage to restore sex hormone balance	Preclinical (*In vivo*)	Ameliorates endocrine dysregulation and IR	(205)
	Formononetin	NLRP3 inflammasome	NLRP3 inflammasome inhibition	Inhibits NLRP3 inflammasome to reduce pro-inflammatory cytokines and oxidative stress	Preclinical (*In vivo*) and Preclinical (*In vitro*)	Alleviates hyperandrogenism and ovarian pathology	(128)
	Crateva religiosa extract	CYP19A1 and PPARγ genes	Aromatase and lipid metabolic regulation	Upregulates CYP19A1 to restore hormones and activates PPARγ to ameliorate IR/oxidative stress	Preclinical (*In vivo*)	Corrects hormonal imbalance and polycystic ovarian morphology	(206)
	Sumac powder	Lipid peroxidation and inflammatory microenvironment	Anti-inflammatory and antioxidant pathways	Decreases inflammatory markers, attenuates lipid peroxidation, and optimizes glycemic/lipid control	Clinical Trial (Phase I/II/III)	Ameliorates hyperandrogenism and cardiometabolic risks	(207)
	C-Phycocyanin	NRF2	NRF2/NLRP3/GSDMD and NRF2-mediated ferroptosis	Prevents granulosa cell pyroptosis and ferroptosis	Preclinical (*In vivo*) and Preclinical (*In vitro*)	Delays PCOS and preserves fertility	(126, 127)
	Green tea extract	Ovarian inflammation and oxidative stress	Catechin-mediated antioxidant and anti-inflammatory mechanisms	Scavenges free radicals, reduces pro-inflammatory cytokines, and modulates metabolic signaling	Preclinical (*In vivo*) and Preclinical (*In vitro*)	Improves metabolic parameters, hormones, and ovarian function	(156)
Food-Derived & Botanical Extracts	Concentrated pomegranate juice	Inflammatory markers and oxidative stress network	Direct antioxidant and systemic anti-inflammatory pathways	Downregulates systemic inflammatory markers and oxidative stress	Clinical Trial (Phase I/II/III)	Ameliorates systemic inflammation and hormonal abnormalities	(208)
	Saffron petal extract and anthocyanin	Steroidogenic enzymes and local immune microenvironment	Inflammatory gene and antioxidant factor modulation	Regulates steroidogenic enzymes, inhibits inflammatory genes, and elevates antioxidant activity	Preclinical (*In vivo*)	Reverses hyperandrogenism-induced ovarian decline	(209)
	Plantago ovata extract	Glucose/lipid metabolism and oxidative stress	Multi-target metabolic regulation	Enhances insulin sensitivity and mitigates oxidative damage	Preclinical (*In vivo*)	Alleviates metabolic syndrome and reproductive endocrine abnormalities	(135)
	Spermidine	NRF2 and Akt	NRF2/HO-1/GPX4 and Akt/FHC/ACSL4	Activates NRF2/Akt axes to suppress localized oxidative stress and ferroptosis	Preclinical (*In vivo*) and Preclinical (*In vitro*)	Attenuates ovarian damage	(210)
Endogenous Molecules、Metabolites, and Vitamins	Melatonin	Circadian rhythm and oxidative stress network	Exosome/miRNA-mediated and antioxidant pathways	Restores circadian rhythms, enhances antioxidant capacity, and reduces apoptosis	Approved/Clinical Guideline	Ameliorates cardiometabolic risks and rescues follicular microenvironment.	(41, 93, 211)
	Melatonin and antioxidants	Immune system and oxidative stress	Immune modulation and antioxidant defense	Suppresses immuno-inflammatory responses and oxidative stress	Melatonin: Approved/Clinical Guideline	Remodels endocrine/immune microenvironments to alleviate PCOS and Hashimoto’s thyroiditis	(93)
	Coenzyme Q10	Oocyte mitochondria	Mitochondrial distribution and energy metabolism	Optimizes mitochondrial distribution to reverse meiotic spindle defects	Approved/Clinical Guideline	Promotes maturation, minimizes aneuploidy, and maximizes fertility	(212–214)
	Vitamin D	Androgen synthesis, apoptosis, and endothelial function	Androgen regulation and antioxidant/anti-apoptotic pathways	Reduces androgens, apoptosis, lipid peroxidation, and cardiovascular risks	Approved/Clinical Guideline	Ameliorates hyperandrogenism and cardiometabolic risks, restoring ovulation	(215, 216)
	Carvedilol and clomiphene	NRF2 and NF-κB	NRF2/NF-κB	Modulates antioxidant and anti-inflammatory networks to reduce microenvironmental stress	Approved/Clinical Guideline	Mitigates PCOS pathology and behavioral phenotypes	(10)
Synthetic Drugs and Small Molecules	Rebamipide	Neuroinflammation and ferroptosis	SIRT1/FOXO1/Wnt/β-catenin and associated ferroptosis	Activates SIRT1 to ameliorate iron overload, oxidative stress, and neuroinflammation	Approved/Clinical Guideline	Exerts neuroprotection and alleviates depressive-like behaviors	(217)
	Semaglutide	Ovarian oxidative stress, autophagy, and inflammation	PI3K/AKT/mTOR and AMPK/SIRT1/NF-κB	Reduces oxidative stress and autophagy via PI3K and inflammation via AMPK/SIRT1	Approved/Clinical Guideline	Improves ovarian function and pathology	(36, 218)
	N-Acetyl-L-cysteine	Oxidative stress and Ggt1 deficiency network	ROS scavenging and antioxidant defense	Scavenges free radicals to repair ovarian oxidative damage and lipid dysmetabolism	Approved/Clinical Guideline	Restores ovulation and improves metabolic/reproductive parameters	(219–221)
	GlyNAC	Glutathione synthesis and mitochondrial function	Glutathione biosynthesis and mitochondrial energy metabolism	Corrects glutathione deficiency, scavenges free radicals, and restores mitochondrial oxidation	Clinical Trial (Phase I/II/III)	Ameliorates IR, endothelial dysfunction, and inflammation	(222)
	Tempol	Intestinal oxidative stress and gut microbiota	Gut microbiota-serum metabolite crosstalk	Targets intestinal oxidative stress to remodel gut microbiota and regulate serum metabolites	Preclinical (*In vivo*)	Ameliorates impaired glucose tolerance, hyperandrogenism, and ovarian dysfunction	(25)
	SIRT1 activation	Granulosa cell ferroptosis	SIRT1-associated ferroptosis	Inhibits ferroptosis, lipid peroxidation, and ROS accumulation	Preclinical (*In vivo*)	Improves ovarian function	(124)
Targeted Interventions and Mechanisms	SIRT3 targeting	Endometrial receptivity	SIRT3-associated signaling	Maintains endometrial function	Preclinical (*In vitro*)	Enhances endometrial receptivity and implantation rates	(35)
	SESN2 upregulation	Granulosa cell ferroptosis	PRDX6 K209 lactylation maintenance	Maintains PRDX6 lactylation to inhibit oxidative stress and ferroptosis	Preclinical (*In vivo*) and Preclinical (*In vitro*)	Ameliorates ovarian and granulosa cell dysfunction	(122)
	Lactate metabolism and androgen antagonism	Granulosa cell lactate accumulation	Androgen-induced apoptosis	Blocks lactate accumulation to alleviate acidic stress and apoptosis	Preclinical (*In vivo*) and Preclinical (*In vitro*)	Reverses adverse ovarian histopathology	(62)
	Skeletal muscle antioxidant intervention	Skeletal muscle oxidative stress	Oxidative stress-insulin signaling crosstalk	Attenuates skeletal muscle oxidative stress to repair insulin signaling	Preclinical (*In vivo*) and Preclinical (*In vitro*)	Ameliorates systemic and peripheral IR	(223)
	Broad-spectrum ROS-targeted therapy	ROS homeostasis	ROS-mediated endocrine and metabolic dysfunction	Blocks ROS-induced ovarian damage to restore metabolic equilibrium	Preclinical (*In vivo*)、 Preclinical (*In vitro*) and Clinical Trial (Phase I/II/III)	Improves endocrine, metabolic, and reproductive functions	(224)
	Nutritional supplements	Insulin receptors and systemic oxidative stress	Multiple metabolic regulation and antioxidant networks	Enhances insulin sensitivity, lowers androgens, and scavenges free radicals	Clinical Trial (Phase I/II/III)	Improves metabolic, hormonal, and cardiovascular profiles	(225, 226)
Dietary Supplements and Functional Foods	Prebiotics and synbiotics	Gut microbiota and cardiometabolism	Gut-brain-ovary axis and lipid metabolism	Optimizes gut microbiota to reduce inflammation and improve glycemic/lipid profiles	Clinical Trial (Phase I/II/III)	Ameliorates cardiometabolic parameters and IR	(227)
	Chitosan oligosaccharide	Ovarian granulosa cells	Granulosa cell inflammation and oxidative stress inhibition	Inhibits pro-inflammatory cytokines and repairs oxidative damage in granulosa cells	Preclinical (*In vitro*)	Improves ovarian microenvironment to facilitate follicular development	(228)
	Natural bee products	Reproductive microenvironment and immune system	Holistic antioxidant, anti-inflammatory, and immunomodulatory networks	Protects germ cells from oxidative damage and regulates local immunity	Preclinical (*In vivo*)	Ameliorates anovulation and enhances fertility	(229)

This table outlines diverse therapeutic interventions, encompassing traditional formulas, bioactive phytochemicals, food-derived extracts, endogenous molecules, and synthetic drugs. These agents ameliorate PCOS-associated reproductive microenvironment deterioration and systemic glucolipid dysmetabolism by targeting the oxidative-inflammatory network, mitigating ER stress, preserving mitochondrial and MAMs homeostasis, alongside inhibiting ferroptosis and pyroptosis. The supporting evidence spans *in vitro* and *in vivo* preclinical models, phase I-III clinical trials, and established guidelines.

ART, assisted reproductive technology; ER, endoplasmic reticulum; IVM, *in vitro* maturation; MAMs, mitochondria-associated endoplasmic reticulum membranes; mtROS, mitochondrial reactive oxygen species; PCOS, polycystic ovary syndrome; ROS, reactive oxygen species; SMP, submitochondrial particle.

### 5.2 Comorbidity of PCOS and the liver microenvironment

PCOS is recognized as a profound risk factor for MASLD, independent of obesity. The mechanistic overlap between these two conditions is substantial. General MASLD models indicate that impaired PACS2 expression and PNPLA3 gene variants substantially promote hepatic steatosis (107, 161, 162). In the specific context of PCOS, however, this canonical hepatic lipid accumulation is distinctively aggravated by hyperandrogenism. Excess androgens not only promote *de novo* lipogenesis but also sensitize the liver to the pro-inflammatory microenvironment, creating a PCOS-specific pathogenic layer atop the classic metabolic pathways.

The liver functions as both a target and a catalyst in PCOS metabolic disturbances, facilitating the systemic dissemination of toxicity (163). Hyperandrogenism substantially promotes this cascade. Unlike its protective role against fat accumulation in men, hyperandrogenism in women synergizes with IR to facilitate persistent hepatic steatosis (9, 164, 165). This synergy upregulates lipid synthesis genes and impairs fatty acid oxidation (166). Non-coding RNAs modulate the SREBP1c pathway to promote intracellular lipid droplet accumulation, a burden effectively alleviated by inhibiting the AR/SREBP1 axis (167, 168).

This toxic microenvironment generates profound intermediate phenotypes. Early hepatic lipid deposition disrupts local homeostasis (167, 169). Subcellularly, excessive mTORC1 activation within the TorS syndrome network links these metabolic disorders to liver disease susceptibility (170). Disrupted calcium signaling and physical contacts at MAMs substantially compromise hepatic insulin responsiveness, establishing local IR (106, 171). Chronic organelle stress combined with PEPCK-mediated gluconeogenesis elevates hepatic glucose production (48). Systemic and genetic factors bidirectionally amplify these phenotypes. Adolescent obesity establishes a mutually deteriorating cycle with liver dysfunction (117, 172), while South Asian ethnicity confers an obesity-independent, highly pro-inflammatory metabolic risk (173). Genetic polymorphisms additionally modulate susceptibility; the PNPLA3 p.1148M and rs738409 variants exacerbate lipid accumulation, whereas the MTARC1 minor allele confers protection (161, 166). Local lipotoxicity and oxidative stress originating from ox-LDL and ox-HDL further aggravate this pathology (174).

Specific biomarkers objectively quantify this deterioration. An elevated free androgen index alongside increased alanine aminotransferase (ALT) and aspartate aminotransferase (AST) signifies severe hepatic involvement (175), frequently accompanied by altered stress-induced factors including METRNL (176). Crucially, steatosis-induced oxidative stress downregulates hepatocyte nuclear factor-4a (HNF-4a) to suppress sex hormone-binding globulin (SHBG) synthesis (174, 177). Consequently, reduced SHBG secretion serves as a critical systemic predictor for liver risk (172, 177, 178). Subcellularly, ER stress promotes specific molecular secretome markers, notably MUP1 downregulation and impaired hepatocyte PACS2 expression (65, 107).

Persistent toxic synergy ultimately contributes to severe complications. Organelle collapse and sustained oxidative stress promote extensive hepatocyte apoptosis, a destructive process negatively validated by the protective effects of SNORA73 knockdown (73, 179). HIF-2α-mediated pro-inflammatory macrophage activation facilitates fibrotic cascades (81). Independent of age and body mass index (BMI), PCOS strongly correlates with severe hepatocellular ballooning, increased liver stiffness, and fibrosis (161, 166, 180). This microenvironment distinctively accelerates youth hepatic toxicity, promoting advanced liver fibrosis five years earlier than in healthy cohorts (164, 180).

These cumulative tissue complications establish persistent comorbidities. Epidemiologically, women with PCOS face a BMI-independent 2- to 4-fold increased risk of NAFLD (175, 181). Mendelian randomization suggests a potential causal link between genetically predicted metabolic dysfunction-associated steatotic liver disease (MASLD) and PCOS risk (169). Severe non-alcoholic steatohepatitis (NASH) remains highly prevalent even with normal ALT levels, emerging seven years prematurely in PCOS patients (164, 173). Collectively, these hepatic comorbidities promote distal cardiovascular disease (167, 182).

Targeted therapeutic interventions validate this multidimensional pathology. Modulating Akt2- FOXO1 and YAP/TAZ pathways ameliorates liver fibrosis and inflammation (183), while activating the Ethe1/Keap1/PINK1 autophagy axis alleviates metabolic dysfunction (40). Furthermore, agents including icariin promote hepatic fatty acid oxidation to attenuate lipid accumulation (184), and natural antioxidants including astaxanthin mitigate lipotoxicity (185). Finally, stem cell-derived exosomal miR-21-5p restores glucose homeostasis by inhibiting BTG2 and activating the IRS1/AKT pathway (186).

### 5.3 PCOS and cardiovascular comorbidities

Cardiometabolic progression in PCOS follows a structured pathogenic continuum. Intrinsically, hyperandrogenism impairs microvascular endothelial function by suppressing endothelin B receptor-mediated nitric oxide (NO) production, even in lean, insulin-sensitive individuals (187). Adolescent oligomenorrhea significantly predicts future cardiometabolic risk (177).Chronic endotoxemia (75, 188), vitamin D deficiency (26), and gut dysbiosis-derived extracellular vesicles synergistically amplify these traits to exacerbate systemic oxidative stress and vascular damage (148).

This pathogenic interplay generates specific biomarkers reflecting progressive cellular damage. Elevated oxidized low-density lipoprotein (ox-LDL) (189), upregulated PCSK9 (92, 137), and increased C-reactive protein (CRP) strongly predict future cardiovascular lesions in youth (190). Simultaneously, increased coronary 3-nitrotyrosine and upregulated renal angiotensinogen transcription indicate substantial tissue-level nitrosative stress and aberrant vascular signaling (26, 191). These molecular shifts contribute to subclinical intermediate phenotypes, primarily atherogenic dyslipidemia and chronic low-grade inflammation (9, 141, 192). Vascularly, patients develop elevated coronary artery calcium scores, increased mean arterial pressure (MAP) (193), and impaired brachial artery flow-mediated dilation (FMD) (194, 195). Notably, PCOS predicts increased carotid intima-media thickness (CIMT) more strongly than traditional risk factors including age and body mass index (196).

Unresolved subclinical phenotypes facilitate precipitate localized organ damage and persistent comorbidities. Structural complications encompass early atherosclerosis (AS), metabolic vascular lesions (100), and extensive cardiac inflammation promoting overt ventricular remodeling (92, 98, 137). This systemic disruption can manifest as distinct clinical entities that share overlapping pathophysiological features with PCOS. Essential hypertension emerges as a distinct comorbidity rather than a peripheral symptom, synergistically facilitated by abnormal renal angiotensin system activation and obstructive sleep apnea-induced hypoxia-inducible factor (HIF) abnormalities (191, 197, 198). Despite Mendelian randomization demonstrating no direct genetic causality, coronary artery disease (CAD) remains a prominent comorbidity alongside metabolic dysfunction-associated steatotic liver disease (MASLD) and systemic metabolic syndrome (177, 199).

## 6 Targeted therapeutic strategies and clinical translation of the organelle oxidative-inflammatory network in PCOS

### 6.1 Targeting the endogenous antioxidant defense system

PCOS is characterized by elevated microenvironmental superoxides and compromised endogenous antioxidant pathways (134). This oxidative stress impairs high-density lipoprotein antioxidant capacity, irrespective of obesity (192).Peripheral monocytes reflect this systemic stress. Downregulated inflammatory transcripts in these nutrient-sensitive cells provide a crucial cellular window for evaluating antioxidant therapies (18). Scavenging ROS to restore endogenous defenses plays an important role in mitigating distal cardiovascular complications (143).Centrally, the transcription factor NRF2 orchestrates this defense network by co-activating heme oxygenase-1 and GPX4, thereby attenuating lipid peroxidation-induced granulosa cell death (125).Therapeutically, endogenous compounds like spermidine activate the NRF2 axis to ameliorate local oxidative stress (210). In addition, biomimetic nanocarriers facilitate NRF2 nuclear translocation to reconstruct ovarian defenses, while carvedilol-clomiphene synergism similarly reshapes this network (10, 114). Beyond NRF2, the Sirtuin family exhibits translational potential for ameliorating reproductive endocrine and oocyte developmental disorders (230). Activated SIRT1 promotes mitochondrial biogenesis to protect cells (33, 125), and inhibits ovarian granulosa cell ferroptosis (124). Via this SIRT1 pathway, edaravone protects damaged cells, ribavirin mitigates lipid peroxidation (217), and somatropin clears ovarian ROS while improving autophagic defects (36, 218). Moreover, mitochondrial SIRT3 counteracts microenvironmental damage by protecting endometrial receptivity and clearing intracellular ROS (35). As a novel antioxidant target, maintaining PRDX6 lactylation serves as a key mechanism through which SESN2 exerts anti-apoptotic protection (122).

Direct exogenous antioxidant supplementation remains a core clinical strategy against systemic oxidative stress (**Table 2**). N-acetylcysteine (NAC) activates defense systems to alleviate gene-deletion-induced reproductive defects and reduces androgen-associated mitochondrial damage alongside local lactate accumulation (62, 219, 220). When combined with taurine or metformin, NAC attenuates systemic oxidative stress and improves ovulatory and clinical outcomes (93, 221, 223, 224). Melatonin enhances antioxidant capacity and helps realign endocrine-disrupted circadian rhythms (41, 211). It alleviates pathology by inhibiting the COX-2 and NF-κB pathways to improve the follicular microenvironment (93, 231, 232). Coenzyme Q10 optimizes oocyte mitochondrial distribution and minimizes *in vitro* maturation aneuploidy (212–214). Meta-analyses indicate that composite supplements containing α-lipoic acid, vitamins, or prebiotics remodel the microbiome to support vascular endothelial function (11, 225, 226), whereas synbiotics further improve cardiometabolic parameters (227). Additionally, chito-oligosaccharides and bee products alleviate granulocyte inflammation and glycemic abnormalities (228, 229).

**Table 2:** Therapeutic interventions targeting oxidative stress and programmed cell death in PCOS.

Category	Intervention	Target	Pathway	Molecular & cellular effects	Evidence level/stage	Clinical outcomes	Reference
Traditional Formulas	Yangjing Zhongyu Tang	Multi-organ ferroptosis	Ferroptosis inhibition	Suppresses multi-organ ferroptosis	Approved/Clinical Guideline	Alleviates PCOS phenotypes	(117)
	Qi Gong Wan	Adipose tissue and metabolic stress	NRF2/HO-1/Cyp1b1	Attenuates adipocyte hypertrophy and oxidative stress to reduce inflammation	Approved/Clinical Guideline	Ameliorates metabolic and reproductive dysfunction	(200)
	Bu-Shen-Tian-Jing Formula	Oxidative stress and glucose metabolism	Mitochondrial SIRT3 signaling	Restores mitochondrial function and regulates oxidative stress and glucose dysmetabolism	Approved/Clinical Guideline	Alleviates PCOS pathogenesis	(201)
Specific Bioactive Phytochemicals	Curcumin and aerobic exercise	ER stress	Hyperandrogenism-induced IRE1α/XBP1	Suppressing hyperandrogenism-induced ER stress. This curtails reactive oxygen species, restores mitochondrial potential, and abrogates granulosa cell apoptosis.	Preclinical (*In vivo*) and Preclinical (*In vitro*)	Ameliorates follicular dysfunction	(203)
	Curcumin and metformin	IR and oxidative stress	NRF2 and antioxidant interaction networks	Enhances insulin sensitivity and inhibits oxidative damage	Metformin: Approved/Clinical Guideline •	Ameliorates reproductive and metabolic disorders	(204)
	Platycodin D	CD44 and granulosa cell ferroptosis	CD44-mediated ferroptosis inhibition	Upregulates CD44 to restore SLC7A11/GPX4,attenuating ferroptosis and oxidative damage	Preclinical (*In vivo*) and Preclinical (*In vitro*)	Ameliorates ovarian injury and follicular maldevelopment	(118)
	Leonurine	Granulosa cell ferroptosis and gut microbiota	SLC7A11/GPX4 axis and gut microbiota crosstalk	Activates SLC7A11/GPX4 to inhibit ferroptosis and regulate gut microbiota	Preclinical (*In vivo*) and Preclinical (*In vitro*)	Improves ovarian function, hormonal balance, and IR	(129)
	Marjoram	Insulin sensitivity and hormonal balance	Inflammation and oxidative stress blockade	Suppresses inflammation and oxidative damage to restore sex hormone balance	Preclinical (*In vivo*)	Ameliorates endocrine dysregulation and IR	(205)
	Formononetin	NLRP3 inflammasome	NLRP3 inflammasome inhibition	Inhibits NLRP3 inflammasome to reduce pro-inflammatory cytokines and oxidative stress	Preclinical (*In vivo*) and Preclinical (*In vitro*)	Alleviates hyperandrogenism and ovarian pathology	(128)
	Crateva religiosa extract	CYP19A1 and PPARγ genes	Aromatase and lipid metabolic regulation	Upregulates CYP19A1 to restore hormones and activates PPARγ to ameliorate IR/oxidative stress	Preclinical (*In vivo*)	Corrects hormonal imbalance and polycystic ovarian morphology	(206)
	Sumac powder	Lipid peroxidation and inflammatory microenvironment	Anti-inflammatory and antioxidant pathways	Decreases inflammatory markers, attenuates lipid peroxidation,and optimizes glycemic/lipid control	Clinical Trial (Phase I/II/III)	Ameliorates hyperandrogenism and cardiometabolic risks	(207)
	C-Phycocyanin	NRF2	NRF2/NLRP3/GSDMD and NRF2-mediated ferroptosis	Prevents granulosa cell pyroptosis and ferroptosis	Preclinical (*In vivo*) and Preclinical (*In vitro*)	Delays PCOS and preserves fertility	(126, 127)
	Green tea extract	Ovarian inflammation and oxidative stress	Catechin-mediated antioxidant and anti-inflammatory mechanisms	Scavenges free radicals, reduces pro-inflammatory cytokines, and modulates metabolic signaling	Preclinical (*In vivo*) and Preclinical (*In vitro*)	Improves metabolic parameters, hormones, and ovarian function	(156)
	Concentrated pomegranate juice	Inflammatory markers and oxidative stress network	Direct antioxidant and systemic anti-inflammatory pathways	Downregulates systemic inflammatory markers and oxidative stress	Clinical Trial (Phase I/II/III)	Ameliorates systemic inflammation and hormonal abnormalities	(208)
Food-Derived & Botanical Extracts	Saffron petal extract and anthocyanin	Steroidogenic enzymes and local immune microenvironment	Inflammatory gene and antioxidant factor modulation	Regulates steroidogenic enzymes, inhibits inflammatory genes, and elevates antioxidant activity	Preclinical (*In vivo*)	Reverses hyperandrogenism-induced ovarian decline	(209)
	Plantago ovata extract	Glucose/lipid metabolism and oxidative stress	Multi-target metabolic regulation	Enhances insulin sensitivity and mitigates oxidative damage	Preclinical (*In vivo*)	Alleviates metabolic syndrome and reproductive endocrine abnormalities	(135)
	Spermidine	NRF2 and Akt	NRF2/HO-1/GPX4 and Akt/FHC/ACSL4	Activates NRF2/Akt axes to suppress localized oxidative stress and ferroptosis	Preclinical (*In vivo*) and Preclinical (*In vitro*)	Attenuates ovarian damage	(210)
	Melatonin	Circadian rhythm and oxidative stress network	Exosome/miRNA-mediated and antioxidant pathways	Restores circadian rhythms, enhances antioxidant capacity, and reduces apoptosis	Approved/Clinical Guideline	Ameliorates cardiometabolic risks and rescues follicular microenvironment.	(41, 93, 211)
Endogenous Molecules、Metabolites, and Vitamins	Melatonin and antioxidants	Immune system and oxidative stress	Immune modulation and antioxidant defense	Suppresses immuno-inflammatory responses and oxidative stress	Melatonin: Approved/Clinical Guideline	Remodels endocrine/immune microenvironments to alleviate PCOS and Hashimoto’s thyroiditis	(93)
	Coenzyme Q10	Oocyte mitochondria	Mitochondrial distribution and energy metabolism	Optimizes mitochondrial distribution to reverse meiotic spindle defects	Approved/Clinical Guideline	Promotes maturation, minimizes aneuploidy, and maximizes fertility	(212–214)
	Vitamin D	Androgen synthesis, apoptosis, and endothelial function	Androgen regulation and antioxidant/anti-apoptotic pathways	Reduces androgens, apoptosis, lipid peroxidation,and cardiovascular risks	Approved/Clinical Guideline	Ameliorates hyperandrogenism and cardiometabolic risks,restoring ovulation	(215, 216)
	Carvedilol and clomiphene	NRF2 and NF-κB	NRF2/NF-κB	Modulates antioxidant and anti-inflammatory networks to reduce microenvironmental stress	Approved/Clinical Guideline	Mitigates PCOS pathology and behavioral phenotypes	(10)
	Rebamipide	Neuroinflammation and ferroptosis	SIRT1/FOXO1/Wnt/β-catenin and associated ferroptosis	Activates SIRT1 to ameliorate iron overload, oxidative stress, and neuroinflammation	Approved/Clinical Guideline	Exerts neuroprotection and alleviates depressive-like behaviors	(217)
Synthetic Drugs and Small Molecules	Semaglutide	Ovarian oxidative stress, autophagy, and inflammation	PI3K/AKT/mTOR and AMPK/SIRT1/NF-κB	Reduces oxidative stress and autophagy via PI3K and inflammation via AMPK/SIRT1	Approved/Clinical Guideline	Improves ovarian function and pathology	(36, 218)
	N-Acetyl-L-cysteine	Oxidative stress and Ggt1 deficiency network	ROS scavenging and antioxidant defense	Scavenges free radicals to repair ovarian oxidative damage and lipid dysmetabolism	Approved/Clinical Guideline	Restores ovulation and improves metabolic/reproductive parameters	(219–221)
	GlyNAC	Glutathione synthesis and mitochondrial function	Glutathione biosynthesis and mitochondrial energy metabolism	Corrects glutathione deficiency, scavenges free radicals, and restores mitochondrial oxidation	Clinical Trial (Phase I/II/III)	Ameliorates IR, endothelial dysfunction, and inflammation	(222)
	Tempol	Intestinal oxidative stress and gut microbiota	Gut microbiota-serum metabolite crosstalk	Targets intestinal oxidative stress to remodel gut microbiota and regulate serum metabolites	Preclinical (*In vivo*)	Ameliorates impaired glucose tolerance, hyperandrogenism, and ovarian dysfunction	(25)
	SIRT1 activation	Granulosa cell ferroptosis	SIRT1-associated ferroptosis	Inhibits ferroptosis, lipid peroxidation,and ROS accumulation	Preclinical (*In vivo*)	Improves ovarian function	(124)
Targeted Interventions and Mechanisms	SESN2 upregulation	Granulosa cell ferroptosis	PRDX6 K209 lactylation maintenance	Maintains PRDX6 lactylation to inhibit oxidative stress and ferroptosis	Preclinical (*In vivo*) and Preclinical (*In vitro*)	Ameliorates ovarian and granulosa cell dysfunction	(122)
	Lactate metabolism and androgen antagonism	Granulosa cell lactate accumulation	Androgen-induced apoptosis	Blocks lactate accumulation to alleviate acidic stress and apoptosis	Preclinical (*In vivo*) and Preclinical (*In vitro*)	Reverses adverse ovarian histopathology	(62)
	Skeletal muscle antioxidant intervention	Skeletal muscle oxidative stress	Oxidative stress-insulin signaling crosstalk	Attenuates skeletal muscle oxidative stress to repair insulin signaling	Preclinical (*In vivo*) and Preclinical (*In vitro*)	Ameliorates systemic and peripheral IR	(223)
	Broad-spectrum ROS-targeted therapy	ROS homeostasis	ROS-mediated endocrine and metabolic dysfunction	Blocks ROS-induced ovarian damage to restore metabolic equilibrium	Preclinical (*In vivo*)、 Preclinical (*In vitro*) and Clinical Trial (Phase I/II/III)	Improves endocrine, metabolic, and reproductive functions	(224)
Dietary Supplements and Functional Foods	Natural bee products	Reproductive microenvironment and immune system	Holistic antioxidant, anti-inflammatory, and immunomodulatory networks	Protects germ cells from oxidative damage and regulates local immunity	Preclinical (*In vivo*)	Ameliorates anovulation and enhances fertility	(229)
	Nutritional supplements	Insulin receptors and systemic oxidative stress	Multiple metabolic regulation and antioxidant networks	Enhances insulin sensitivity, lowers androgens, and scavenges free radicals	Clinical Trial (Phase I/II/III)	Improves metabolic, hormonal, and cardiovascular profiles	(225, 226)
	Prebiotics and synbiotics	Gut microbiota and cardiometabolism	Gut-brain-ovary axis and lipid metabolism	Optimizes gut microbiota to reduce inflammation and improve glycemic/lipid profiles	Clinical Trial (Phase I/II/III)	Ameliorates cardiometabolic parameters and IR	(227)

This table systematically summarizes potential therapeutic interventions aimed at mitigating oxidative stress and programmed cell death in PCOS. Selected for pathophysiological relevance and methodological rigor, this table rank therapies across a four-tier clinical evidence hierarchy, systematically excluding unverified *in vitro*-only interventions.

ACSL4, acyl-CoA synthetase long-chain family member 4; Akt, protein kinase B; AMPK, AMP-activated protein kinase; CD44, cluster of differentiation 44; CYP19A1, cytochrome P450 family 19 subfamily A member 1; Cyp1b1, cytochrome P450 family 1 subfamily B member 1; ER, endoplasmic reticulum; FHC, ferritin heavy chain; FOXO1, forkhead box O1; Ggt1, gamma-glutamyltransferase 1; GlyNAC, glycine and N-acetylcysteine; GPX4, glutathione peroxidase 4; GSDMD, gasdermin D; HO-1, heme oxygenase-1; IR, insulin resistance; IRE1α, inositol-requiring enzyme 1 alpha; miRNA, microRNA; mTOR, mechanistic target of rapamycin; NF-κB, nuclear factor kappa B; NLRP3, NLR family pyrin domain containing 3; NRF2, nuclear factor erythroid 2-related factor 2; PCOS, polycystic ovary syndrome; PI3K, phosphoinositide 3-kinase; PPARγ, peroxisome proliferator-activated receptor gamma; PRDX6, peroxiredoxin 6; ROS, reactive oxygen species; SESN2, sestrin 2; SIRT1, sirtuin 1; SIRT3, sirtuin 3; SLC7A11, solute carrier family 7 member 11; XBP1, X-box binding protein 1.

Despite this therapeutic potential, exogenous antioxidant interventions face clinical stratification challenges. For instance, Vitamin D counteracts lipid peroxidation via microenvironmental glutathione modulation. However, its efficacy is contingent upon receptor polymorphisms (215, 216). Clinically, the standalone efficacy of exogenous antioxidants is limited by baseline BMI and metabolic profiles (233). To overcome these limitations, plant-derived drugs and traditional Chinese medicine (TCM) offer a multi-target paradigm for ameliorating systemic redox imbalances. Traditional formulas systematically modulate organelle defense systems. For example, Yangjing Zhongyu Tang activates the NRF2 axis to ameliorate local oxidative stress (117), and Qigong Wan attenuates adipose tissue hypertrophy via this pathway (200). Classical kidney-tonifying formulas ameliorate metabolic and reproductive impairments via the SIRT3 system (201). Natural botanical extracts exhibit profound organelle-protective properties. Phycocyanin attenuates ovarian ferroptosis and granulosa cell pyroptosis (126, 127). Curcumin reshapes mitochondrial biogenesis via SIRT1/PGC-1α upregulation and inhibits ER stress (132, 204, 234). Specific plant derivatives, notably prunetin, platycodin D, and leonurine, inhibit inflammasomes and modulate ferroptosis (118, 128, 129).

Systemically, concentrated pomegranate juice, green tea extract, Qiliutong combined with silver nanoparticles, and lacquer tree powder scavenge free radicals to downregulate inflammatory markers (156, 207, 208, 235). Simultaneously, extracts from saffron, marjoram, plantain seed, and fishwood improve ovarian endocrine function and local steroid synthases (135, 205, 206, 209). By supporting endogenous glutathione networks and reshaping the gut microbiota-metabolite interactome (25, 222), these botanical agents and traditional prescriptions provide a promising framework for comprehensive PCOS management (**Table 2**). Dietary interventions and bioactive compounds constitute foundational non-pharmacological therapies for PCOS. Aligned with the Food and Medicine Homology (FMH) paradigm, these strategies help mitigate metabolic dysregulation, oxidative stress, and organelle dysfunction (236). Specifically, botanical extracts confer systemic multi-target protection against diet-induced metabolic toxicities by modulating gut microbiota homeostasis and corresponding extracellular vesicles (237). Translational evidence from classic FMH agents including Panax ginseng demonstrates that phytochemicals substantially reinforce endogenous antioxidant defenses, attenuate organelle-derived DAMP signaling, and support metabolic-reproductive homeostasis (238).

### 6.2 Cutting-edge explorations in organelle-targeted therapies

Overcoming conv-entional pharmacological limitations in PCOS necessitates subcellularly targeted therapies (146). Metabolically, inner-membrane-absorbed MitoQ indirectly curtails ROS by effectively blocking lipid peroxidation cascades (52). While independent of conventional insulin signaling, it significantly enhances skeletal muscle glucose uptake under lipotoxicity (52). Additionally, the lipophilic nature of melatonin facilitates organelle entry, establishing it as a promising natural mitochondria-targeted antioxidant (54).

Mitigating protein misfolding and ER stress constitutes a vital therapeutic axis. TUDCA suppresses inflammatory cytokines and pyroptosis, while restoring granulosa cell mitochondrial function by inhibiting IRE1 phosphorylation (15, 17). Pathway-specific inhibitions similarly confer organelle protection; blocking Notch signaling via the γ-secretase inhibitor DAPT attenuates ER stress-mediated cumulus cell expansion arrest (239). Targeted inhibition of the ER-associated adipocyte protein PDIA4 ameliorates obesity-related metabolic comorbidities (71). Exogenous recombinant MUP1 supplementation selectively decelerates hepatic protein synthesis, substantially alleviating lipotoxic ER burden (65). Regarding organelle communication, activating the NO/sGC/PKG pathway preserves MAM functional integrity, whereas STX17 inhibition blocks aberrant interactions and mitigates calcium overload (104, 106).

Modulating autophagy and abrogating programmed cell death offer another critical dimension of organelle-targeted therapy. Specific ferroptosis inhibitors, effectively Fer-1, ameliorate microscopic ovarian damage and preserve fertility (55, 126). Systemically, this targeted intervention ameliorates impaired glucose tolerance, ovulatory dysfunction, and hyperandrogenism *in vivo* (130). Similarly, rosastat mitigates ferroptosis, emerging as a promising cell death-modulating strategy (22). Regarding autophagy, non-pharmacological modalities like traditional acupuncture alleviate organelle damage by downregulating granulosa cell LncMEG3 expression and inhibiting the mTOR pathway (67, 155). Formulations notably Guizhi-Fuling-Wan substantially restore reproductive function by modulating autophagic flux (240).

Finally, cutting-edge nanomedicine and extracellular vesicle (EV) delivery systems hold preliminary potential of cell-free, organelle-level precision therapy. Lipid nanoparticle-mediated localized ovarian delivery of NCOA7 mRNA selectively enhances lysosomal function and stress granule clearance, highlighting the translational potential of microscopic genetic interventions (64). Engineered macrophage membrane-camouflaged biomimetic nanocarriers achieve immune evasion and precise targeted accumulation within the oxidatively damaged ovarian microenvironment (114). Drug-free nanozyme systems utilizing cerium oxide nanoparticles, alongside resveratrol complexes, effectively scavenge local oxidative stress to ameliorate ovarian inflammation (95). Alternatively, adipose-derived mesenchymal stem cell exosomes substantially ameliorate anovulation and hyperandrogenemia, establishing a promising foundation for non-pharmacological bioengineering therapies (146, 186). This strategy substantially reshapes systemic metabolism by promoting mitophagy; moreover, naturally derived bacterial EVs can traverse tissue barriers, serving as novel exploratory avenues for targeted drug delivery (83, 148) (**Table 3**).

**Table 3:** Targeted PCOS modulators: organelle homeostasis, epigenetic networks, and intercellular crosstalk.

Intervention	Target	Pathway	Molecular & cellular effects	Evidence level/stage	Clinical outcomes	Reference
MitoQ	Inner mitochondrial membrane	Lipid peroxidation cascade blockade	Blocks inner membrane lipid peroxidation to reduce ROS	Clinical Trial (Phase I/II/III)	Enhances insulin-independent skeletal muscle glucose uptake during lipotoxicity	(52)
Melatonin	Mitochondria	Lipophilic organelle targeting and Clock gene regulation	Acts as a lipophilic antioxidant and regulates Clock genes to restore organelle homeostasis	Preclinical (*In vivo*) and Preclinical (*In vitro*)	Eliminates mitochondrial oxidative stress	(54)
Anti-androgen or Anti-pyroptosis interventions	Ovarian ER	Inflammation and pyroptosis blockade	Suppresses ER stress and blocks pyroptosis	Preclinical (*In vivo*) and Preclinical (*In vitro*)	Reduces ovarian inflammation to reverse follicular dysplasia	(15)
ER stress or Notch blockers	ER and Notch	Notch signaling regulation	Inhibits ER stress-induced aberrant Notch activation	Preclinical (*In vivo*) and Preclinical (*In vitro*)	Restores cumulus-oocyte complex expansion to enhance oocyte quality	(239)
PDIA4 inhibitors	Adipose tissue ER	ER stress buffering	Inhibits PDIA4 to reduce adipose inflammation and upregulate adiponectin	Preclinical (*In vivo*) and Preclinical (*In vitro*)	Reverses obesity-related metabolic comorbidities	(71)
Exogenous recombinant MUP1	Hepatic protein synthesis	Lipotoxic stress mitigation	Decelerates hepatic protein synthesis	Preclinical (*In vivo*) and Preclinical (*In vitro*)	Reduces lipotoxic ER burden	(65)
STX17 inhibitors	STX17 and MAMs	PARKIN-MCUb aberrant interaction blockade	Disrupts aberrant ER-mitochondria contacts to mitigate calcium overload	Preclinical (*In vivo*) and Preclinical (*In vitro*)	Ameliorates obesity-related metabolic and organelle stress	(104, 106)
NO/sGC/PKG pathway activators	MAMs	Inter-organelle communication protection	Activates NO/sGC/PKG signaling to preserve MAM integrity	Preclinical (*In vivo*) and Preclinical (*In vitro*)	Activates NO/sGC/PKG signaling to preserve MAM integrity	(104, 106)
Fer-1	Lipid peroxidation network	Precision ferroptosis blockade	Blocks hyperandrogenism-induced granulosa cell ferroptosis	Preclinical (*In vivo*) and Preclinical (*In vitro*)	Reverses ovarian damage to preserve fertility	(55)
C-Phycocyanin	Ferroptosis system	NRF2-mediated anti-ferroptosis	Activates NRF2 pathway to scavenge lipid peroxides	Preclinical (*In vivo*) and Preclinical (*In vitro*)	Prevents ovarian damage to rescue fertility	(126)
Roxadustat	EGLN1-HIF1α-ferroptosis axis	Proteomic targeted blockade	Targets EGLN1 and HIF1α to inhibit ferroptosis	Preclinical (*In vivo*) and Preclinical (*In vitro*)	Improves PCOS phenotypes	(130)
Acupuncture	Ovarian granulosa cell autophagy	LncMEG3-mediated PI3K/AKT/mTOR inhibition	Downregulates LncMEG3 to inhibit PI3K/AKT/mTOR and modulate autophagy	Preclinical (*In vivo*) and Preclinical (*In vitro*)	Ameliorates follicular and ovulatory dysfunction	(22)
Electroacupuncture	Skeletal muscle and systemic autophagy	Autophagy enhancement and organelle homeostasis	Enhances autophagy to alleviate mitochondrial and ER stress	Preclinical (*In vivo*)	Improves IR and systemic metabolic syndrome	(155)
Guizhi Fuling Wan	Ovarian granulosa cell autophagy	PI3K/AKT/mTOR restoration	Restores PI3K/AKT/mTOR signaling to suppress excessive granulosa cell autophagy	Preclinical (*In vivo*)	Ameliorates IR and PCOS pathology	(67, 240)
NCOA7	Stress granules and V-ATPase	V-ATPase interaction and stress granule clearance	Accelerates clearance of aberrant stress granules to mitigate organelle stress	Preclinical (*In vivo*) and Preclinical (*In vitro*)	Alleviates ovarian microenvironment aging	(64)
Macrophage biomimetic quercetin nanocarriers	Ovarian lesions and local ROS microenvironment	Macrophage targeting and ROS-responsive release	Enables ROS-responsive targeted drug release	Preclinical (*In vivo*) and Preclinical (*In vitro*)	Ameliorates structural ovarian pathology	(114)
Cerium nanocomposites	Ovarian immune microenvironment and macrophages	M2 macrophage polarization and antioxidation	Exerts nanoscale antioxidant catalysis to skew macrophage polarization toward the M2 phenotype macrophage polarization	Preclinical (*In vivo*) and Preclinical (*In vitro*)	Reverses immune dysregulation to alleviate endocrine/inflammatory damage	(95)
AMSC-EXOs	Hepatic metabolic network and BTG2	miR-21-5p/IRS1-AKT activation	Transfers miR-21-5p to inhibit BTG2 and activate metabolic signaling	Preclinical (*In vivo*) and Preclinical (*In vitro*)	Reverses polycystic phenotypes and metabolic disorders to enhance fertility	(186)
GMEVs	Gut-brain-ovary axis and host metabolic system	Gut microecology cross-domain regulation	Delivers microbial molecules to remodel host metabolism and inflammation.	Preclinical (*In vivo*) and Preclinical (*In vitro*)	Improves PCOS metabolism, IR, and hyperandrogenism	(148)

This table outlines advanced targeted modulators acting on deep cellular mechanisms, specifically focusing on organelle homeostasis, epigenetic remodeling, and intercellular crosstalk in PCOS pathogenesis. Selected for pathophysiological relevance and methodological rigor, this table rank therapies across a four-tier clinical evidence hierarchy, systematically excluding unverified *in vitro*-only interventions.

AKT, protein kinase B; AMSC-EXOs, amniotic/adipose mesenchymal stem cell-derived exosomes; BTG2, B-cell translocation gene 2; EGLN1, egl-9 family hypoxia inducible factor 1; ER, endoplasmic reticulum; Fer-1, ferrostatin-1; GMEVs, gut microbiota-derived extracellular vesicles; HIF1α, hypoxia-inducible factor 1-alpha; IR, insulin resistance; IRS1, insulin receptor substrate 1; LncMEG3, long non-coding RNA maternally expressed gene 3; MAMs, mitochondria-associated membranes; MCUb, mitochondrial calcium uniporter dominant negative beta subunit; miR-21-5p, microRNA-21-5p; MitoQ, mitoquinone; mTOR, mechanistic target of rapamycin; MUP1, major urinary protein 1; NCOA7, nuclear receptor coactivator 7; NO, nitric oxide; NRF2, nuclear factor erythroid 2-related factor 2; PCOS, polycystic ovary syndrome; PDIA4, protein disulfide isomerase family A member 4; PI3K, phosphoinositide 3-kinase; PKG, protein kinase G; ROS, reactive oxygen species; sGC, soluble guanylate cyclase; STX17, syntaxin 17; V-ATPase, vacuolar-type ATPase.

### 6.3 Mechanisms of deep organelle regulation in existing metabolic drugs

Metformin, a classical metabolic agent, transcends glycemic control by comprehensively reshaping organelle homeostasis and immune-inflammatory networks via AMPK activation (241, 242). Mitochondrially, it selectively inhibits hepatocyte respiratory complex I, reducing NADH oxidation, inner membrane proton gradients, and oxygen consumption (49). It restores hepatic mitophagy via the Ethe1/Keap1/PINK1 axis (40) and normalizes ATP production in PCOS-derived stem cells (61, 243). Antagonizing programmed cell death, metformin specifically inactivates the p38 MAPK pathway to profoundly suppress granulosa cell ER stress (71, 244). It rescues trophoblasts from ferroptosis (82, 116). Systemically, it attenuates placental STAT3 phosphorylation and modulates circulating superoxide dismutase 1, significantly ameliorating the oxidative microenvironment (90, 245). Crucially, metformin profoundly suppresses ovarian macrophage pyroptosis by enriching gut Akkermansia (99). Ultimately, these multidimensional mechanisms provide a robust pharmacological foundation for microenvironmental repair and cardiovascular risk reduction (246, 247).

Emerging anti-diabetics, notably GLP-1 receptor agonists, potently reshape deep cellular networks beyond weight loss (248). Liraglutide downregulates coronary microcirculatory Nox-1 and endothelin-1, conferring systemic cardioprotection by inhibiting NF-κB and IL-1β (249). Semaglutide restores ovarian autophagy via the PI3K/AKT/mTOR pathway (36) and attenuates severe local inflammation via the AMPK/SIRT1/NF-κB axis (218). Exenatide similarly modulates systemic adipokine and pro-inflammatory cytokine homeostasis (241). Advancing these strategies, combining the dual agonist tirzepatide with adropin mitigates intramyocardial metabolic inflammation via the AKT/GSK3β/NLRP3 pathway, microscopically reversing myocardial pathology and repairing striated structures (137). Ultimately, glucagon receptor triple agonists further broaden therapeutic avenues for targeting complex comorbidities and cellular metabolic substrate reorganization (248).

SGLT2 inhibitors and established metabolic modulators also exhibit novel organelle-targeting mechanisms. Independent of macroscopic IR, SGLT2 inhibitors significantly attenuate white adipose oxidative stress, directly restoring impaired mitochondrial function (250). They exert crucial microscopic protection by specifically inhibiting cardiomyocyte sodium-hydrogen exchanger 1, maintaining transmembrane ionic homeostasis (250). The DPP-4 inhibitor sitagliptin remodels impaired post-transcriptional inflammatory and extracellular matrix networks (37), whereas linagliptin effectively suppresses high-glucose-induced apoptotic misfolded protein responses and ER stress in ovaries (74). The antioxidant α-lipoic acid profoundly reshapes mitochondrial energy metabolism in damaged tissues by enhancing Akt activation and upregulating the PGC-1α axis (60). Similarly, the traditional agent berberine augments glucose uptake by promoting specific SIRT3 ubiquitination and degradation (251), while specifically inhibiting hyaluronan synthase 2 to alleviate systemic low-grade inflammation (4, 252). Ultimately, repurposing existing metabolic drugs constitutes a core strategy for comprehensively restoring target-organ homeostasis via deep regulation of ER stress and mitochondrial networks.

## 7 Current limitations and future perspectives

Despite significant insights into organelle stress and immunometabolic reprogramming in PCOS, critical limitations persist. Foremost, generalized pathophysiological models obscure marked clinical and biochemical heterogeneity. Under the Rotterdam criteria, PCOS encompasses four distinct clinical phenotypes defined by varying degrees of hyperandrogenism, ovulatory dysfunction, and polycystic ovarian morphology. Proposed subcellular cascades, including mitochondrial fragmentation, chronic ER stress, mitochondria-associated membrane distortion, and lipid peroxidation-linked ferroptosis, are closely interplay with synergistic hyperandrogenism and IR. These organelle-centric mechanisms exhibit high generalizability primarily to classic metabolic and hyperandrogenic phenotypes. Conversely, in non-hyperandrogenic or lean normo-insulinemic patients, organelle damage may be secondary to alternative neuroendocrine or intra-ovarian signaling pathways. Therefore, universal extrapolation of these subcellular findings across all PCOS subtypes warrants caution.

Targeted therapeutics such as mitochondrial antioxidants and stem cell-derived exosomes rely predominantly on preclinical and *in vitro* data. Validating their translational efficacy and long-term safety demands rigorous, phenotype-stratified prospective cohorts to definitively exclude off-target toxicity affecting oocyte viability and offspring health. Ultimately, current mechanistic frameworks fail to completely bridge microscopic pathology with macroscopic clinical heterogeneity. Realizing true precision medicine necessitates constructing multidimensional network models that integrate environmental endocrine disruptors, the gut-organ axis, and multi-omic transgenerational epigenetics.

## 8 Methods

A systematic literature search was conducted to identify relevant studies investigating the organelle oxidative-inflammatory network, cellular death pathways, and metabolic phenotypes in PCOS. The primary databases queried included PubMed, Web of Science, Embase, and Scopus, covering literature from their respective inceptions up to February 2026.

The search strategy employed combinations of Medical Subject Headings (MeSH) and free-text keywords, utilizing Boolean operators (AND/OR). The core search strings were structured as follows: (“Polycystic Ovary Syndrome” OR “PCOS”) AND (“organelle” OR “mitochondrial dysfunction” OR “endoplasmic reticulum stress” OR “mitochondria-associated membranes” OR “MAMs”) AND (“oxidative stress” OR “inflammation” OR “pyroptosis” OR “ferroptosis” OR “autophagy” OR “apoptosis”) AND (“metabolic dysfunction” OR “insulin resistance” OR “hyperandrogenism”).

To ensure the rigor and relevance of the synthesized evidence, inclusion criteria were restricted to: (1) peer-reviewed original research articles (*in vitro*, *in vivo* animal models, and human clinical studies); (2) high-impact review articles providing necessary theoretical context; and (3) publications written in the English language. Emphasis was placed on studies published within the last 10 years to capture the most recent advancements in multi-omics and subcellular mechanisms.

Articles were excluded if they were: (1) non-peer-reviewed preprints; (2) conference abstracts without full-text availability; (3) case reports; or (4) studies lacking a clear focus on the subcellular or molecular mechanisms of PCOS. After removing duplicates, the titles and abstracts were independently screened, and the full texts of potentially eligible articles were reviewed for final inclusion in this comprehensive narrative review.

## 9 Conclusion

PCOS pathogenesis represents a systemic convergence of metabolic toxicity, organelle failure, and immune-inflammatory dysregulation. The collapse of mitochondrial and ER homeostasis, triggered by an oxidative-inflammatory microenvironment, induces multi-organ injury and non-apoptotic programmed cell death. The persistent systemic dissemination of DAMPs and extracellular vesicles facilitates irreversible metabolic and cardiovascular deterioration. Consequently, clinical paradigms must transition toward precision metabolic modulation and organelle-targeted therapeutics. Restoring organelle homeostasis through ER stress mitigators, mitochondria-targeted antioxidants, and advanced nano-delivery systems to block lipid peroxidation offers a transformative strategy to reverse the comprehensive pathological sequelae of PCOS.
